# The Era of Gene Therapy: The Advancement of Lentiviral Vectors and Their Pseudotyping

**DOI:** 10.3390/v17081036

**Published:** 2025-07-24

**Authors:** Bat-Erdene Jargalsaikhan, Masanaga Muto, Masatsugu Ema

**Affiliations:** 1 Department of Stem Cells and Human Disease Models, Research Center for Animal Life Science, Shiga University of Medical Science, Seta, Tsukinowa-cho, Otsu 520-2192, Japan; mmuto@belle.shiga-med.ac.jp (M.M.); mema@belle.shiga-med.ac.jp (M.E.); 2 Medical Genome Center, National Cerebral and Cardiovascular Center, 6-1 Kishibe-Shinmachi, Suita 564-8565, Japan; 3 Institute for the Advanced Study of Human Biology (ASHBi), Kyoto University, Yoshida-Konoe-cho, Sakyo-ku, Kyoto 606-8501, Japan

**Keywords:** gene therapy, lentiviral vectors, pseudotyping, envelope proteins

## Abstract

Over 35 years of history, the field of gene therapy has undergone much progress. The initial concept—the replacement of dysfunctional genes with correct ones—has advanced to the next stage and reached the level of precise genome editing. Dozens of gene therapy products based on viral and non-viral delivery platforms have been approved, marking the dawn of the gene therapy era. These viral vector strategies rely on adenoviruses, adeno-associated viruses, lentivirus-derived tools, and so on. From the middle of the gene therapy transition, despite the challenges and serious negative consequences, the lentiviral vector has emerged as a cornerstone and demonstrated benefits in fields ranging from basic science to gene therapy. Therefore, we outline the importance of lentiviral vectors in the gene therapy era by focusing on their roles in the clinical usage, derivation, and development of next-generation platforms, as well as their pseudotyping.

## 1. The Derivation of Lentiviral Vectors

**Figure 1:**
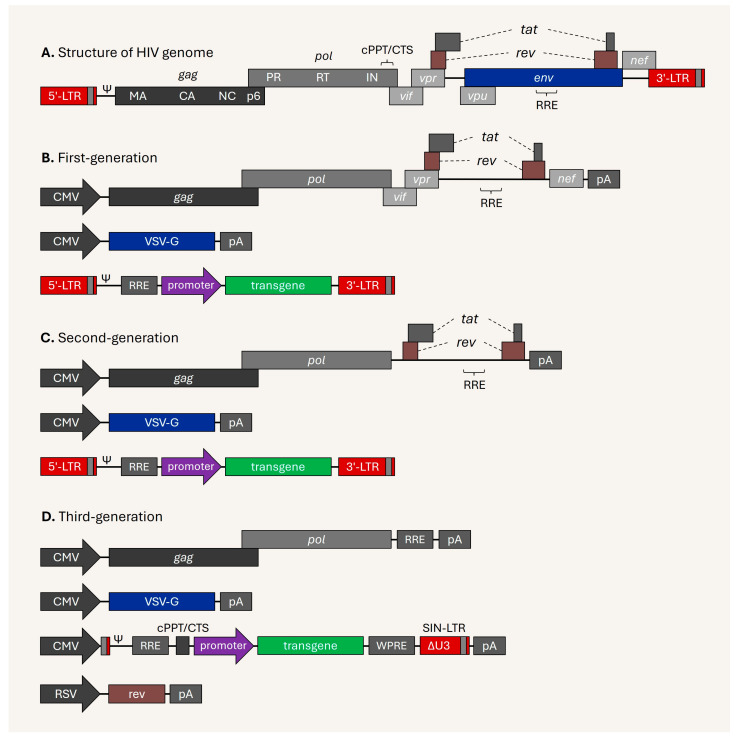
Schematic illustration of HIV-1 genome and three generations of lentiviral vectors. (**A**) The HIV-1 genome is flanked by an LTR that consists of U3 (long red), R (gray), and U5 (short red) regions, encoding structural (*gag, gag-pol,* and *env*), regulatory (*rev* and *tat*), and accessory (*vif, vpr, vpu,* and *nef*) protein-coding genes. The packaging signal (ψ) is required to package vRNA into the viral particle. The Gag precursor contains the core viral proteins—including the matrix (MA), capsid (CA), nucleocapsid (NC), and p6 proteins—while the Gag-Pol precursor additionally contains the protease (PR), reverse transcriptase (RT), and integrase (IN) proteins. (**B**) First-generation vector system. The packaging plasmid includes all HIV-1 genes in a single vector except for *env* and *vpu*. The separated envelope plasmid provides envelope protein. The transfer plasmid contains a transgene expression cassette flanked by the HIV-1 LTRs. (**C**) Second-generation vector system. The packaging plasmid contains all HIV-1 genes in a single vector without accessory protein sequences. The separated envelope plasmid provides envelope protein. The transfer plasmid contains a transgene expression cassette flanked by the HIV-1 LTRs. (**D**) Third-generation vector system. The packaging plasmid contains only the *gag-pol* sequence with RRE element. The separated envelope plasmid provides envelope protein. The transfer plasmid includes CMV promoter-driven truncated 5′-LTR, required *cis*-acting elements, transgene expression cassette, WPRE element and partially deleted self-inactivating ΔU3-LTR (SIN-LTR) with polyadenylation (pA), and separated Rev expression plasmid vector.

Acquired immunodeficiency syndrome (AIDS) was first recognized as a new disease in 1981 [1]. Luc Montagnier and his colleagues soon discovered that the cause of AIDS is a novel virus in the Retroviridae family, which was officially named human immunodeficiency virus (HIV) in 1986 [2]. HIV-1 and HIV-2, the main types of HIV, are positive-sense single-stranded RNA (ssRNA) viruses that contain reverse transcriptase (RT). The HIV-1 genome is approximately 9.7 kb and contains several *cis*-acting elements and 9 open reading frames that allow it to produce three main polyprotein precursors (Gag, Gag-Pol, and envelope), two regulatory proteins (Tat and Rev), and four accessory (Vif, Vpr, Vpu, and Nef) proteins [3,4] (Figure 1A).

An MLV-derived gamma-retroviral vector from the Retroviridae family was first developed in the early 1980s, based on transient transfection of separated plasmid vectors into producer cells [5,6], which showed the ability to transduce dividing cells and integrate transgenes into the host genome [7]. It can be pseudotyped using heterologous envelope proteins, such as vesicular stomatitis virus glycoprotein G (VSV-G), resulting in a high titer and broad tropism [8]. The gamma-retroviral vector was first introduced into gene therapy trials in the 1990s [9,10].

The first-generation LV system was developed in 1996 by Naldini and colleagues using the same principles as those used for the gamma-retroviral vectors as above, and is capable of transducing both dividing and non-dividing cells [11], offering exciting new potential for inserting genes into the genome of non-proliferating cells. The system contains three plasmid vectors, as illustrated in Figure 1B: (I) the packaging plasmid encodes all of the proteins excluding the HIV envelope protein and Vpu accessory protein, with the HIV-1 packaging signal and *cis*-acting elements deleted from the untranslated region; (II) the envelope plasmid encodes VSV-G protein or heterologous envelope protein; and (III) the transfer plasmid carries the transgene expression cassette under an internal promoter flanked by the HIV-1 long terminal repeat (LTR), containing the HIV-1 packaging signal and required *cis*-elements [11].

The second-generation LV system was subsequently developed in 1997 by Zufferey and colleagues [12], in which sequences encoding the accessory proteins Vif, Vpr, Vpu, and Nef were eliminated (Figure 1C). Although these accessory proteins confer a survival advantage with respect to lentivirus replication in vivo, they are not vital for viral growth in vitro [12,13].

Just a year after that, third-generation LV systems were reported in 1998 about the same time by Miyoshi [14] and Dull [15] and their respective colleagues. In this generation, (I) regulatory and accessory proteins were completely eliminated from the packaging plasmid vector; (II) the envelope plasmid encodes the VSV-G protein or heterologous envelope protein; (III) the transfer vector contains the CMV promoter-driven truncated 5′-LTR, required *cis*-acting elements, transgene expression cassette, partially deleted self-inactivating ΔU3-LTR (SIN-LTR) and polyadenylation (pA) sequences; and (IV) a separated regulatory plasmid vector which expresses only the Rev protein. A 133–400 bp deletion in the promoter and enhancer region of the 3′-LTR enables self-inactivation by eliminating LTR-driven gene expression in the integrated provirus [14,15]. During reverse transcription, the ΔU3-LTR is transferred to the 5′-LTR, allowing the proviral DNA to be flanked by the ΔU3-LTR [14,15,16]. Moreover, the U3 region of the 5′-LTR was replaced with a strong heterologous constitutive promoter (RSV or CMV) in the transfer plasmid vector, providing Tat-independent vRNA transcription during production [14,15,16]. The SIN-LTR reduces the risks of vector mobilization, replication-competent vectors, and unintended activation of nearby genes at the integration site [17,18,19]. The third-generation system eventually became a versatile tool [20], following the subsequent improvement of the transfer vector construct: first, through inclusion of the post-transcriptional regulatory element of woodchuck hepatitis virus (WPRE) before the self-inactivating LTR (SIN-LTR) [21]; and second, through insertion of the central polypurine tract/central termination sequence (cPPT/CTS) between the Rev-responsive element (RRE) and an internal promoter sequence [22] (Figure 1D). The WPRE significantly improves gene expression by enhancing mRNA processing and export in both intronless and spliced mRNAs, regardless of the cell cycle state of the transduced cell [21]. Nuclear localization of the DNA flap after reverse transcription of the viral genome is dependent on the cPPT/CTS, allowing the lentiviral vector to efficiently infect the host cells—especially non-dividing cells [22,23]—when compared with the MLV-derived gamma-retroviral vector that relies on mitosis [24]. Another attractive and useful aspect is integrase-defective lentiviral vectors (IDLVs). Several integrase-defective mutants at functional domains of IN, such as class I (e.g., D64V, D116N, E152A) and class II (e.g., D167K, D167A, Q168A, 262RRK), have been reported and investigated, reducing proviral integration [25,26,27,28]. Mutations on IN that primarily diminish the integration step are categorized as class I, while class II mutants mainly hamper particle assembly, reverse transcription, and nuclear import of the pre-integration complex (PIC) [25,26,27,28]. The D64V variant had reduced integration activity by up to 1/10,000 compared to the wild-type virus [25], unintegrated vectors maintain transient gene expression from episomal DNA up to several months, and the D64V is most common variant regarding the usage of IDLVs [20,26,27,28]. Finally, the third-generation lentiviral vector system, with a capacity of up to 10 kb cargo, is now considered a cornerstone in gene and protein delivery technology, having been utilized from basic science to gene therapy [20].

## 2. Clinical Use of Lentiviral Vectors

The first Phase 1 lentiviral vector clinical trial was conducted in 2003, which assessed the safety of autologous CD4+ T cells that were ex vivo transduced using an HIV-1-derived lentiviral vector encoding a 937-base antisense sequence against the HIV envelope. The trial involved five patients with HIV-1 who had previously experienced treatment failures with antiretroviral therapy. Among the participants, three patients demonstrated significant reductions in viral load, along with stable or increased CD4+ T cell counts [29,30]. Additionally, for all 65 subjects in Phases 1 and 2, no adverse events related to the therapy were recorded [31]. Over the past two decades, lentiviral vectors have emerged as powerful tool and there have been remarkable advances in the clinical application of such vectors, highlighting their enormous potential for gene therapy aimed at a wide range of inherited and acquired diseases.

**Figure 2:**
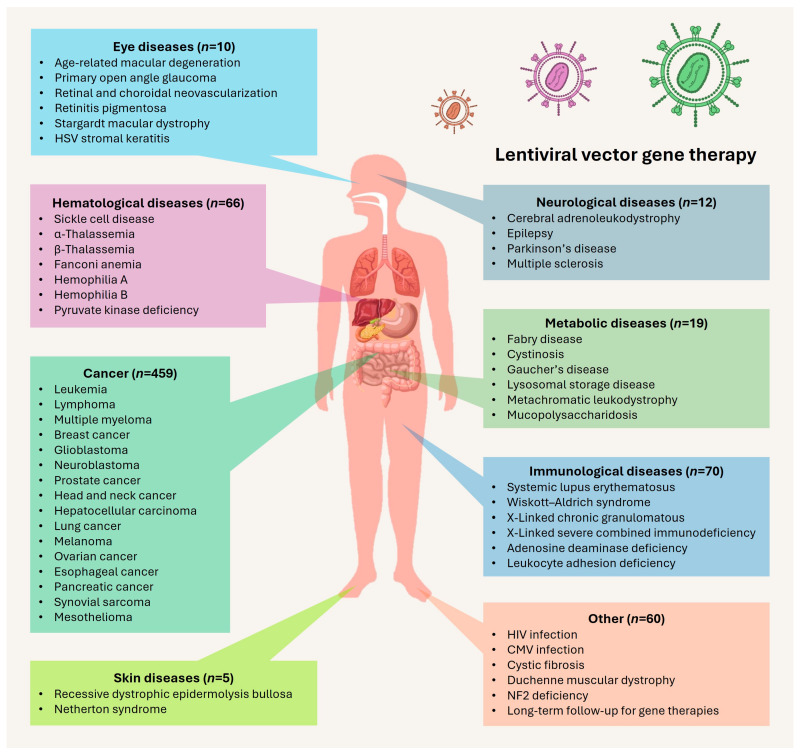
Clinical applications of lentiviral vectors in major human diseases. The applications cover various diseases—including neurological, ocular, hematological, metabolic, cancer, immunological, skin, viral infection, and lung diseases—as well as long-term follow-up for the lentiviral vector gene therapy, as detailed in Supplementary Table S1.

We comprehensively summarized 701 registered clinical trials of the lentiviral vector-based gene therapies shown in Supplementary Table S1. Of those, 209 clinical trials involved lentiviral vector-mediated gene transfer therapies, with lentivirus-like particles used to deliver CRISPR/Cas in ocular diseases in 4 clinical trial registries, 44 trials for T cell receptor (TCR) cell therapy, and the rest of the registries involving chimeric antigen receptor (CAR) cell therapy. Overall, the clinical applications of lentiviral vectors can be divided into five main uses: (I) correction of faulty or mutated genes by delivering a functional copy to ex vivo HSCs, (II) delivery of CAR or TCR to the ex vivo T cells, (III) in vivo direct gene transfer, (IV) antisense or shRNA delivery, and (V) delivery of gene editing tools, indicating broad-range utilization of lentiviral vectors in the field. The major human diseases in clinical trials include neurological, ocular, hematological, metabolic, cancer, immunological, skin, viral infection, and lung diseases, as well as long-term follow-up for viral vector gene therapies (Figure 2).

Gene therapy—especially the cutting-edge approaches of CAR-T and TCR-T cell therapy—has recently achieved remarkable success in treating cancers that have failed to respond to conventional treatments [32,33,34]. The concept is that genetic modification of T cells can increase their ability to specifically target cancer cells [35,36]. CAR-T or TCR-T cell therapy involves collecting T cells from patients or donors, selecting a suitable subclass (e.g., Treg, CD3, CD4, CD8, CD4/CD8), and performing ex vivo genetic modification by transducing them with a lentiviral vector carrying CAR gene or TCR gene expression cassette, followed by expansion and quality control of the transduced cells, as well as cryopreservation of the CAR-T or TCR-T cells. The cryopreserved cells can be stored for up to several months, ready for infusion into the patients to fight against cancer [37,38]. CAR-T directly targets cancer cells via CAR, which consists of a cancer antigen-specific external single-chain variable fragment (scFv), a transmembrane domain, an intracellular signaling domain, and a costimulatory domain from receptors (e.g., CD28, OX40, CD137), while TCR-T recognizes intracellular specific antigens presented by major histocompatibility complex (MHC) molecules of cancer cells through the transduced natural TCR (α- and β-chains non-covalently linked to the CD3 complex on the surface), which leads to the activation of T cells and destruction of cancer cells [39,40].

TCR-T cell therapy is considered valuable against solid tumors. For example, it has been shown to be effective in melanoma by targeting the New York esophageal squamous cell carcinoma (NY-ESO-1) antigen [41]; effective for synovial sarcoma, ovarian cancer, and head and neck cancer treatment by recognizing melanoma antigen gene A4 (MAGE-A4) antigen [42,43]; and promising against hepatocellular carcinoma (HCC) via identification of alpha-fetoprotein (AFP) antigen [44]. Moreover, mesothelin (MESO)-targeting TCR-T cell therapy is encouraging and is under clinical trials for various cancers including mesothelioma, lung cancer, ovarian cancer, and other solid tumors [45].

CAR-T cell therapy has demonstrated outstanding performance in treating hematologic malignancies, with approximately 80% malignancy response of complete or partial remission [46]. Several CAR-T gene therapy products are available that recognize and destroy CD19-positive B cell malignancies, which are notable for treating specific B-cell non-Hodgkin lymphomas, B-cell ALL, and CLL [47,48,49,50,51,52,53]. Furthermore, plasma cell-associated protein B-cell maturation antigen (BCMA)-specific CAR-T cells are effective in the treatment of multiple myeloma [54,55,56,57,58]. In clinical trials, CAR-T cell therapies targeting various tumor-associated antigens (TAAs), such as CD19/CD20, C-type lectin-like molecule-1 (CLL-1), CD22, CD133, and CD171, have been assessed [59,60,61,62,63,64].

Lentiviral vector-mediated gene transfer into CD34+ HSCs has been utilized in the treatment of several genetic disorders, including X-linked severe combined immunodeficiency (X-SCID) caused by mutations in the IL2RG gene [65]; sickle cell disease [66] and β-thalassemia [67], both resulting from mutations in the HBB gene; Fanconi anemia caused by mutations in the FANCA gene [68]; metachromatic leukodystrophy (MLD) due to mutations in the ARSA gene [69]; cerebral adrenoleukodystrophy (CALD) associated with mutations in the ABCD1 gene [70]; hemophilia A arising from mutations in the F8 gene [71]; and Wiskott–Aldrich Syndrome (WAS), which is caused by mutations in the WAS gene [72].

Moreover, lentiviral vector-based gene therapy products directly injected into the target tissue include tyrosine hydroxylase (TH), aromatic L-amino acid decarboxylase (AADC), and GTP-cyclohydrolase 1 (CH1) gene delivery in brain striatum for Parkinson’s disease [73]; endostatin and angiostatin gene transfer via subretinal injection to treat neovascular age-related macular degeneration (AMD) [74]; and ABCA4 gene delivery to the eyes of Stargardt Disease patients [75], all resulting in well-tolerated and sustained transgene expression.

Despite these successful stories, lentiviral vector-mediated gene therapy presents some adverse effects, such as cytokine release syndrome, neurotoxicity, severe infection, and prolonged cytopenia in the short-term as well as a theoretical risk of insertional secondary malignancy in the long-term [76]. Besides, in vivo CRISPR/Cas-mediated gene editing therapy trials have utilized lentivirus-like particles to treat ocular diseases, including primary open-angle glaucoma with MYOC gene mutations and refractory herpetic viral keratitis [77], expanding their potential.

## 3. The Gene Therapy Era and Lentiviral Vectors

**Figure 3:**
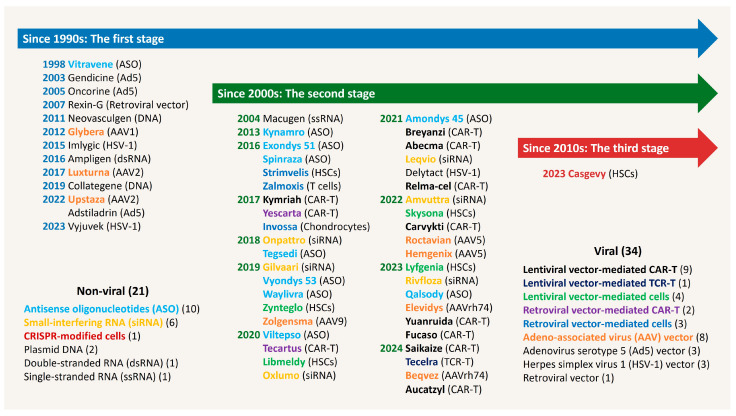
Developmental stages of the gene therapy era and approved products. The first-stage gene therapy products originated from the first-generation delivery platforms and oligonucleotides. The second-stage products are based on second-generation or more advanced delivery platforms and oligonucleotides. The third-stage product includes precise genome editing tools.

The United States (US) Food and Drug Administration (FDA) broadly defines human gene therapy as a therapeutic technique that modifies or manipulates the expression of a gene or alters the biological properties of living cells. The guidelines also include the use of gene therapy products to achieve a therapeutic or preventive effect via the transcription or translation of transferred genetic material or altering human genetic sequences [78]. According to the regulatory document of the European Medicines Agency (EMA), gene therapy medicinal products contain recombinant nucleic acids or a substance used to regulate, repair, replace, add, or delete a genetic sequence for therapeutic, preventive, or diagnostic purposes. Its effect is directly related to the genetic sequence or its expression product [79]. Both the FDA and EMA guidelines widely consider recombinant nucleic acids (e.g., plasmids DNA, RNA), genetically modified microorganisms (e.g., viruses, bacteria, fungi), engineered site-specific nucleases, and ex vivo genetically modified human cells to be human gene therapy products, except for vaccines against infectious diseases [78,79]. At the end of 2024, 55 gene therapy products had been approved [47,48,49,50,51,52,53,55,56,57,58,66,67,69,70,80,81,82,83,84,85,86,87,88,89,90,91,92,93,94,95,96,97,98,99,100,101,102,103,104,105,106,107,108,109,110,111,112,113,114,115,116,117,118,119,120], excluding non-genetically engineered cell therapy and vector vaccines. These have been categorized into three developmental stages, as shown in Figure 3.

In 1989, Rosenberg and his colleagues demonstrated the feasibility of using autologous human cells transduced with a gamma-retroviral vector derived from murine leukemia virus (MLV) for gene therapy [10], which marked the beginning of the gene therapy era. The original concept of gene therapy was to treat hereditary disorders caused by faulty genes by delivering a functional copy of the gene to the affected cells using non-viral and viral vectors [121,122,123]. Therefore, the first therapeutic clinical trial involving the autologous infusion of T cells with the gamma-retroviral vector-mediated adenosine deaminase (ADA) gene transfer was conducted in two children with Severe Combined Immunodeficiency due to adenosine deaminase deficiency (ADA-SCID) monogenic disease in 1990 [9,124]. Gene therapy holds great promise and, since then, hundreds of gene therapy clinical trials have been conducted worldwide using potential viral and non-viral gene delivery methods, laying the foundation for further development. For instance, several clinical trials were conducted to introduce a functional copy of the cystic fibrosis membrane conductance regulator (CFTR) gene into the airway cells of patients with cystic fibrosis caused by CFTR mutations in the early 1990s [125,126,127,128,129]. These trials used different tools for therapeutic purposes. In the UK and US, researchers have used cationic liposomes combined with complementary DNA [125,126]. In the US and France, a human Adenovirus serotype 5 (Ad5) viral vector has been used [127,128]. In the US, a human Adeno-associated virus serotype 2 (AAV2) viral vector [129] was also used to transfer a functional copy of the CFTR gene into the airway epithelium. However, these attempts did not provide sustained lung function for cystic fibrosis patients due to ineffective gene transfer and the inhibition of AAV2, cationic lipofection, and Ad5 via bronchial secretion and local immune responses [130,131,132]. Unfortunately, in 1999, the first notorious adverse effect of gene therapy was the death of an 18-year-old male with partial ornithine transcarbamylase (OTC) deficiency, due to a severe immune reaction after an intra-arterial infusion of human adenovirus type 5, deleted in E1 and E4, containing human OTC cDNA [133]. Later, at the end of 2002, patients treated for X-linked SCID (X-SCID) using the ex vivo genetically modified autologous CD34+ hematopoietic stem cells (HSCs) with MLV-derived gamma-retroviral vector-based transfer of IL2RG expression cassette started to develop lymphoid T-cell leukemia [134]. This gene therapy-related secondary malignancy was caused by insertional activation of the proto-oncogenes LMO-2, BMI1, and CCND2 in almost half of the patients [135,136]. These initial clinical trials revealed serious treatment-related toxicities, including inflammatory responses to the vectors, as well as secondary malignancies resulting from the viral vector-mediated activation of proto-oncogenes; therefore, similar trials were halted by the governments of France and the US [137]. These early attempts—with all the setbacks and lessons learned—can be considered the first stage of human gene therapy development [138].

The second stage of gene therapy was initiated in early 2000s, stimulated by more basic research in virology, immunology, cell biology, model development, and targeted diseases, which led to the development of improved gene therapy products and their successful application in gene therapy [139]. While the UK Gene Therapy Advisory Committee was inviting feedback on the use of the MLV-derived gamma-retroviral vectors—particularly regarding the potential improvements associated with self-inactivating (SIN) vectors as an alternative to gamma-retroviral vectors [137]—the first clinical trial using a human immunodeficiency virus 1 (HIV-1)-derived lentiviral vector was started in January of 2003 in the US, offering a safer and more efficient viral vector [140]. The MLV-derived gamma-retroviral vector integration sites are typically located in the transcription region, while the HIV-1-derived lentiviral vector insertions were significantly reduced in that region and evenly distributed throughout the rest of the gene region [141,142]. Additionally, lentiviral vectors can transduce both dividing and non-dividing cells [143,144], suggesting a safer and more suitable choice than gamma-retroviral vectors. From 2010, the use of the lentiviral vectors in gene therapy clinical trials has increased dramatically, surpassing gamma-retroviral vectors [145]. Furthermore, due to the improved safety achieved by removing enhancer-promoter elements from the LTR, MLV-derived SIN gamma-retroviral vectors for gene therapy are used to this day [48,49,92,93,96] and have been formally approved (Figure 3). Moreover, SIN lentiviral vectors (both integrative and non-integrative) have been used in clinical trials for various therapeutic applications—particularly ex vivo transduction—including in hereditary genetic disease by introducing functional genes to HSCs; in cancers by delivering chimeric antigen receptor (CAR) and tumor specific T-cell receptor (TCR) to the T cells; and in RNA and protein delivery to target cells [20]. Subsequently, the first regulatory approval of a lentiviral vector gene therapy product occurred in 2017; namely, HIV-1-derived lentiviral vector-mediated chimeric antigen receptor (CAR)-T cells targeting CD19 to treat B cell malignancies [146]. In the treatment of X-SCID via ex vivo genetically modified CD34+ HSCs, the usage of SIN lentiviral vector demonstrates benefits over the gamma-retroviral vector, such as providing multilineage engraftment and effective immune reconstitution [65]. Moreover, three decades after the earlier venture to treat cystic fibrosis via gene therapy, the first human trial involving CFTR-targeting therapy with a lentiviral vector began in 2024, utilizing a Simian immunodeficiency virus (SIV)-derived lentiviral vector pseudotyped with Sendai virus (SeV) fusion (F) and hemagglutinin-neuraminidase (HN) envelope proteins [147]. The SeV-F/HN pseudotyped SIV-derived lentiviral vector is ideal for introducing transgenes into airway cells due to its high affinity for α2,3 sialylated N-acetyllactosamine (LacNAc) [148,149].

In the case of AAV vectors, the discovery of natural serotypes beyond AAV5 (such as AAV8, 9, and rh74), the development of engineered capsids that evade immune responses, and the design of vectors for prolonged transgene expression and improved safety have led to the next generation, with AAV gene therapy products having received official approval since 2012 [150]. Furthermore, the earliest antisense oligonucleotides (ASOs) that entered clinical trials consisted of a phosphodiester or phosphorothioate backbone without any modifications [151]. They were transformed with modifications including 2′-O-Methyl (2′-O-Me), 2′-O-Methoxyethyl (2′-O-MOE), and 2′-Fluoro (2′-F), as well as chemical modification of the furanose ring with next-generation oligonucleotides (ASO and siRNA), consequently demonstrating clinical efficacy and safety and also gaining approval [152,153]. ASOs are single-stranded synthetic oligonucleotides, typically 15-30 bases long, designed to bind the target RNA (pre-mRNA or mRNA) to regulate gene expression through the mechanism of either RNase H-mediated RNA degradation or altering its processing [81,153,154]. In contrast, siRNAs—which are typically 20-25 bp long double-stranded RNA molecules (modified or non-modified)—lose one strand (the passenger strand) after Dicer cleavage in the cytoplasm, while the remaining strand (the guide strand) leads to target mRNA degradation by forming an RISC (RNA-induced silencing complex) [81,152,154]. Further, potential viral and non-viral methods for gene transfer are still being explored, such as a novel Anellovector that is under development from the human native Anelloviridae viruses, which do not cause any known diseases [155]. The majority of gene therapy products have advanced to the next generation at this stage and have reached official approval, offering safer and more effective approaches for the delivery of genetic material (DNA or RNA) or proteins into human cells for therapeutic purposes.

From the 2010s, the potential for the precise replacement or editing of mutated genes with exact corrections appeared with the achievement and development of admirable gene editing tools based on engineered site-specific nucleases, such as Zinc-finger nuclease (ZNF) [156], Transcription Activator-Like Effector Nuclease (TALEN) [157,158], and Clustered Regularly Interspaced Short Palindromic Repeats (CRISPR)/CRISPR-associated protein (Cas) (CRISPR/Cas) [159]. Moreover, site-specific nuclease-based next-generational base editing [160] and prime editing [161] provide tools to edit DNA without double-strand break, which evolved promptly and play a crucial role in the field [162,163]. After only a short period, the CRISPR/Cas genetically modified cell therapy astonishingly received official approval in 2023 [164]. Additionally, gene therapy needs to be highly effective, with minimal toxicity and low cost [46]. Delivering the CRISPR/Cas system to target cells is challenging, and one of the most important delivery tools in both in vitro and in vivo applications is lentiviral vectors [165,166,167]. Essentially, lentiviral vectors can be used to deliver CRISPR/Cas to cells in two main ways. First, integrative and non-integrative lentiviral vectors that simply carry CRISPR/Cas and sgRNA expression cassettes have become an important tool in biomedical research [167,168]. Another method is to utilize lentivirus-like particles that are packaged with engineered site-specific nucleases or cargo proteins [166,169,170,171]. Current in vivo gene editing therapy trials employ lentivirus-like particles to deliver the CRISPR/Cas system [77], suggesting the continued potential of recombinant lentiviral vectors.

Gene therapy is believed to be the medicine of the 21st century [172] and tremendous progress has been made in recent years, with 6,333 clinical trials including a range of promising treatments for a variety of diseases registered by the end of 2024 [173]. Additionally, 21 non-viral and 34 viral gene therapy products have been approved (Figure 3); for a detailed overview, see Supplementary Table S2. Notably, 14 are lentiviral vector gene therapies, highlighting their significance within the area. Lentiviral vectors have emerged as precious tools, having entered clinical application from the second-stage of the gene therapy era and demonstrating benefits.

## 4. Current Issues and Development of Next-Generation Lentiviral Vectors

**Figure 4:**
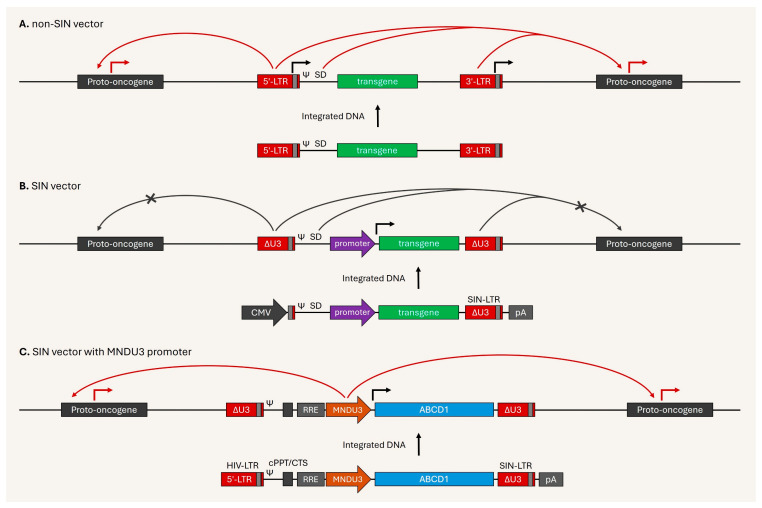
Self-inactivating (SIN) vectors and current concerns. (**A**) Non-SIN vectors flanked by full LTRs in the integrated provirus, each LTR consisting of U3 (long red), R (gray), and U5 (short red) regions. The packaging signal (ψ). The U3 region has an enhancer-promoter sequence. The transcription starting site is the U3 and R junction in 5′-LTR, indicating potential transcriptional leakage on the 3′-LTR. The enhancer-promoter activity in the LTRs and possible splicing donor (SD) acceptor events are prone to activating nearby genes at the integrated sites. (**B**) The deletion of the enhancer-promoter sequence in 3′-U3 (ΔU3) allows for SIN-LTR. The SIN vectors are flanked by SIN-LTRs in the integrated provirus, each SIN-LTR consisting of ΔU3 (Δlong red), R (gray), and U5 (short red) regions. Transgene expression is driven by a suitable internal promoter. The loss of enhancer-promoter function in SIN-LTR minimizes the negative impact of the integrated provirus on nearby genes. (**C**) Inclusion of the Myeloproliferative sarcoma virus enhancer, Negative control region deleted (MNDU3) internal promoter to express ABCD1 cDNA in SIN lentiviral vector, leading to activation of nearby genes at the integrated site.

The third-generation lentiviral vector system is still considered state-of-the-art, even two decades after it obtained its current form and began to be utilized in gene therapy [20]. Although CAR-T has achieved remarkable triumphs against blood cancer [54], there remain concerns due to the possibility of insertional oncogenesis [174]. Furthermore, the US FDA has investigated CAR-T therapy-related secondary malignancies and revealed 33 cases among the 30,000 people who had been treated with it, as of the first quarter of 2024 [175]; however, there is a debate regarding whether the danger is due to the original blood cancer or the cutting-edge therapy. However, the benefits outweighed the risks and saved thousands of lives [176]. In particular, deleting the enhancer and promoter sequences from the LTR, SIN gamma-retroviral, and lentiviral vectors minimized the risk of unintended activation of nearby promoters at the integration sites [17,18,19] (Figure 4A,B).

In hundreds of clinical trials over two decades using lentiviral vectors for gene therapy, no serious complications had been reported until recently. Unfortunately, 7 of 67 patients who suffered CALD—a rare genetic disorder that damages the brain and spinal cord caused by ABCD1 gene mutation—developed hematological malignancies after infusion of lentiviral vector-mediated ex vivo genetically modified autologous HSCs [177]. The issue is likely due to activation of the proto-oncogenes near the integration sites through the Myeloproliferative sarcoma virus enhancer, Negative control region deleted (MNDU3) internal promoter in the SIN lentiviral vector that drives ABCD1 cDNA expression [178], as shown in Figure 4C. The lentiviral vectors—which used human cellular gene promoters in at least 16 other diseases—have shown a strong safety record over the past 15 years [178], suggesting that vector design is vital. Additionally, the MND-LTR promoter had stronger and more persistent transgene expression in hematopoietic cells of all lineages compared to the MLV-LTR promoter [179]. Usage of the LTR promoter-enhancer derived from gamma retroviruses as an internal promoter in the SIN lentiviral vector, as well as inclusion of the gamma-retroviral LTR promoter-enhancer at the LTR sequence of the lentiviral vector, can lead to increased activation of nearby genes, potentially contributing to tumorigenic processes [180,181].

**Figure 5:**
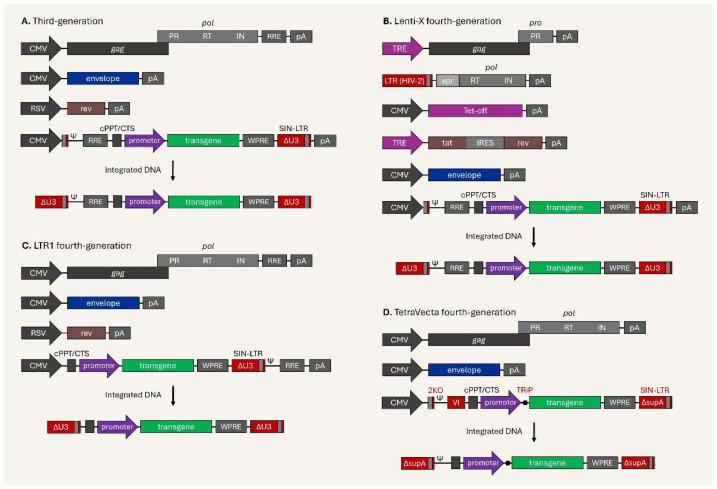
Schematic illustration of third-generation and next-generation lentiviral vectors. (**A**) Third-generation vector system with 4 plasmid vectors and integrated provirus. (**B**) Lenti-X fourth-generation vector system with 6 plasmid vectors and integrated provirus. (**C**) LTR1 fourth-generation vector system with 4 plasmid vectors and integrated provirus. (**D**) TetraVecta fourth-generation vector system with 3 plasmid vectors and integrated provirus.

Simultaneously, several next-generation lentiviral vector platforms are being developed and/or are ready for deployment. The first next-generation lentiviral vector, named Lenti-X fourth-generation, which has been commercially available since around the end of the 2010s, is a product of Takara Bio Inc (Japan) which has been widely used in basic research [182,183,184,185]. Transfer and envelope plasmid vectors remain intact in the Lenti-X and, in order to produce ultra-high titers of lentiviral vectors, a complicated packaging system was introduced compared with that in the previous generation (Figure 5A,B).

The new system contains Tetracycline-controlled transactivator (tTA), which binds to the tetracycline response element (TRE) in the promoter region, leading to a high level of gene expression in the absence of tetracycline/doxycycline [186]; the tTA is shown as Tet-off under the CMV promoter in Figure 5B. The packaging construct *gag-pol* was divided into two separated plasmids—(I) the TRE promoter-driven *gag-pro* expression cassette, and (II) *vpr* accessory and *pol* under LTR from HIV-2 (LTR-2)—ensuring safety and reducing the risk of replication-competent vectors. When the *gag-pro-pol* sequence was divided into two separate components, *gag-pro* and *pol*, the frequency of recombination-dependent DNA mobilization was remarkably reduced when compared with the intact plasmid vector [187]. Moreover, high-titer lentiviral vector production is achieved via intense expression of viral essential proteins through the TRE (can be disabled with Tet-off) and LTR-2 (driven by Tat-transactivator) promoters.

**Figure 6:**
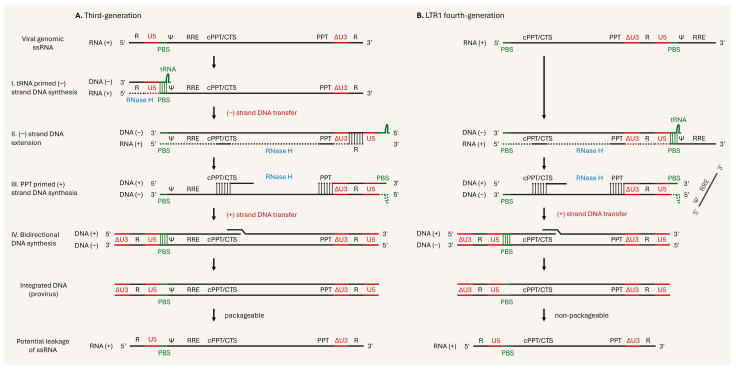
Reverse transcription of lentiviral vectors. (**A**) Reverse transcription with two-strand DNA transfer. Viral genomic (+) ssRNA of the third-generation system is flanked with R elements in both 5′-LTR and 3′-LTR. The required cis-acting elements are located after the 5′-LTR. (I) The binding of tRNA to the primer binding site (PBS) initiates (−) strand DNA synthesis via RT. The RNA-DNA hybrid is degraded by the RNase H activity of RT during the reverse transcription. (II) The (−) strand DNA extension is continued after the (−) strand DNA transfer. The complementary (R) sequence is involved in the first jump. (II-III) All template RNA digestion, including tRNA, except cPPT and PPT, allows for initiation of the PPT-primed (+) strand DNA synthesis. (IV) The annealing of PBS sites transfers the (+) strand DNA, facilitating bidirectional DNA synthesis to continue and complete. The provirus flanked with ΔU3-LTR, containing required cis-acting elements, has risk of potential leakage of packageable vRNA. (**B**) Reverse transcription with single-strand DNA transfer. Viral genomic (+) ssRNA of the LTR1 fourth-generation system starts with PBS and has a single full ΔU3-LTR followed by PBS, the packaging signal (ψ), and RRE. (II) The binding of tRNA to the PBS after the full ΔU3-LTR initiates (−) strand DNA synthesis and extension by RT, without (I) initiation and (−) strand DNA transfer. The RNA–DNA hybrid is degraded by the RNase H activity of RT during reverse transcription. (II–III) All template RNA digestion, including tRNA, except cPPT and PPT, allows for initiation of the PPT-primed (+) strand DNA synthesis, resulting in removal of (ψ) and RRE. (IV) The annealing of PBS sites transfers the (+) strand DNA, enabling bidirectional DNA synthesis to continue and complete. It cannot be packaged in the event of potential leakage of LTR-1 proviral vRNA, due to the elimination of (ψ).

Vink and his colleagues developed the LTR1 fourth-generation lentiviral vector system by rearranging the *cis*-acting elements in the transfer plasmid vector (Figure 5C) that controls the complex replication process of the lentiviral genome in the host, and demonstrated that this next-generation system could produce sufficient titers for preclinical gene therapy in a hemophilia B mouse model [188,189]. The packaging, envelope, and regulatory plasmid vectors were the same as in the previous version. Although SIN vectors were thought to have no LTR-driven transcription due to deletion of the promoter/enhancer elements, full-length packageable vRNA was exhibited from proviral DNA, indicating some risk [190]. While traditional SIN vectors have both a 5′ and 3′ LTR flanking the viral genome, the LTR1 fourth-generation vector features PBS at the 5′ end and a single complete SIN-LTR followed by PBS, the packaging signal (ψ), and RRE at the 3′ end. As a result of this modification, the LTR1 requires only a single DNA transfer jump (unlike the classical two-step DNA transfer jump), enabling removal of the packaging signal (ψ) and RRE during reverse transcription (Figure 6A,B). Hence, even if full-length vRNA leakage occurs in LTR1 provirus, the elimination of the packaging signal (ψ) significantly reduces vector mobilization.

Another novel platform, the TetraVecta System™—fourth-generation lentiviral vector system by Oxford Biomedica (UK) in 2023—is characterized by enhanced safety, quality, and large payload capacity, achieved by introducing up to four systematic modifications on the transfer plasmid [191,192,193,194]. Firstly, the 2KO modification at the major splicing donor (MSD) site, optimized MSD-inactivating sequences, along with a new class of vRNA enhancers based on modified U1 snRNA [192], provides unspliced viral genomic RNA (vgRNA) during production, as HIV-1 splicing at the SD site is dependent on U1 snRNA [195]. The RRE/rev-independent version of the TetraVecta fourth-generation system (illustrated in Figure 5D) that contains the 2KO and synthetic Vector-Intron (VI) features does not rely on the modified U1 snRNA. Instead, the vgRNA is produced by splicing out of the VI, which occurs nearly 100% of the time [194]. Secondly, viral sequences with the RRE element are removed and replaced with the VI, allowing approximately 1 kb of additional space for the payload. The system is Rev-independent and does not need a regulatory plasmid vector [194]. Optionally, a bacterial tryptophan RNA-binding attenuation protein (TRAP) binding sequence overlaps the transcriptional start site of the transgene, and co-expression of TRAP inhibits transgene translation during viral vector production, which is Transgene Repression in Vector production (TRiP) system [196,197]. This is beneficial for the production of lentiviral vectors in CAR-T cells or cytotoxic payloads. The last refinement is bidirectional poly A sequences (supA) at the SIN-LTR, which are 50-fold stronger than the previously used sequences, thus improving transcriptional insulation [191,193].

It is generally known that third-generation lentiviral vector systems adopt roughly 20% of the HIV-1 genome sequence, which integrates into the host genome along with the transgene. Next-generation systems substantially decrease the HIV-1 genome sequence in the integrated provirus, down to approximately 5% for LTR1 and 10% for TetraVecta platforms. As current issues, it has been suggested that vector design and the choice of internal promoter in the SIN vector are critical; however, the fourth-generation systems have unique features and aim for enhanced safety, quality, production efficiency, and payload capacity, as well as minimized viral backbone and toxicity.

## 5. Structure of the Lentiviral Particle and Its Pseudotyping

**Figure 7:**
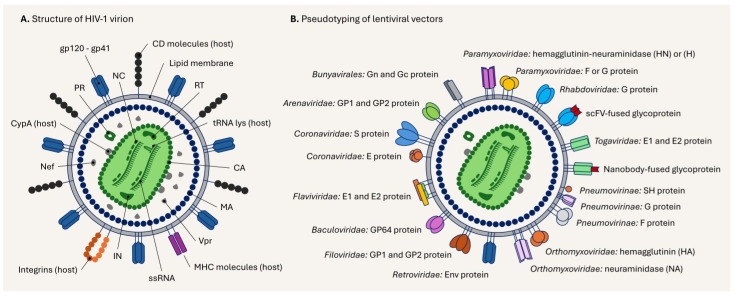
Lentiviral particle structure and its pseudotyping. (**A**) The structure of the mature HIV-1 virion, resembling a lentiviral vector particle. The two copies of viral genomic ssRNA are encapsulated by the capsid (CA) along with the nucleocapsid (NC), reverse transcriptase (RT), integrase (IN), and tRNA lys, which are surrounded by a lipid membrane and matrix protein (MA) with incorporated envelope and receptor proteins. Protease (PR), viral, and host cell proteins are also assembled in the particle. (**B**) The orders, families, or subfamilies of viruses that encode envelope glycoproteins which are employed to pseudotype lentiviral vectors, as well as to engineer envelope proteins.

The mature HIV-1 virion is a spherical particle, approximately 100 nm, surrounded by a lipid membrane with glycoproteins containing a conical capsid, viral genomic ssRNA, and proteins [198]. As the lentiviral vector is derived from HIV-1, it resembles the virion in structure (Figure 7A); however, the incorporation of regulatory and accessory proteins depends on the method of lentiviral vector generation.

The protease (PR) is incorporated into particles as part of the monomer Gag-Pol polyprotein and then dimerizes to become active during viral budding, leading to the maturation of viral particles [199]. The capsid is a protein shell known as p24 that surrounds ribonucleoprotein (RNP) particles and includes two copies of viral genomic ssRNA, ncRNAs (e.g., 7SL RNA [200,201], tRNA lys [202,203]), viral enzymes (IN and RT), and viral nucleocapsid proteins. Cyclophilin A (CypA) is a host cell protein that is incorporated and binds to the HIV-1 capsid [204]. Moreover, cytoskeletal proteins from the host cell—such as actin, myosin, and ezrin—are incorporated into the particle [205]. Although regulatory and accessory proteins could be incorporated, Vpr is a predominant component in the viral particle that is critical for viral replication in target cells [206]. Furthermore, depending on the host cell type, different types of proteins from the host are incorporated into the viral particles to varying degrees [207]. The matrix (MA) protein is inside the lipid membrane, which is crucial for viral replication, maturation, and the structure of the particle [208]. The outer layer of the viral particle is a lipid membrane with envelope proteins. Interestingly, the HIV-1 gp120-gp41 envelope protein is incorporated in the envelope membrane along with cellular molecules such as MHC class I and II, CD molecules (e.g., CD54, CD63, CD11), and integrins (e.g., integrin α4β7) [207,209,210]. The host cell molecules assembled in the envelope play a role in evading the immune response and facilitating cell-to-cell transmission [207,209,210]. The HIV-1 virion enters cells using the envelope proteins in two main ways: (I) direct fusion with the cell membrane [3,4,211,212] or (II) endocytosis and fusion with intracellular compartments [212,213]. Then, the lentiviral genome integrates into the genome of the target cell through a sequential process that reverses transcription via RT and tRNA lys, entering into the nucleus through the nuclear pore complex (NCP), using viral IN and cellular LEDGF/p75 as co-factors [203,214].

It is worth noting that the gp120-gp41 envelope protein can be replaced with heterologous glycoproteins—a common technique recognized as pseudotyping, which is analogous to adapting one’s attire to different contexts [215,216]. The interaction between the envelope protein and the target receptor on the cell membrane determines viral entry, while pseudotyping alters tropism [215,216]. The viral envelope glycoproteins of enveloped viruses can be incorporated into lentiviral vectors through several strategies, including the use of a wild-type, a truncated form, modified cytoplasmic tail, or an engineered envelope protein [148,217,218,219]. Single-envelope protein pseudotyping is the most familiar scenario, such as VSV-G, MLV-A, MLV-E, and RD114 [219]. Additionally, dual or multiple pseudotyping is possible, through which two or more envelope proteins can be incorporated into a single lentiviral particle. Phenotypically unmixed pseudotyping of lentiviral particles is characterized by the incorporation of viral envelope proteins from a single origin, whereas phenotypically mixed pseudotyped lentiviral particles incorporate multiple (usually dual or triple) envelope proteins from distinct origins on the same viral particle surface [217,218,220,221,222]. Table 1 lists representative examples of pseudotypes exhibiting phenotypically unmixed and mixed characteristics. The heterologous envelope proteins are well adopted in lentiviral vectors for phenotypically unmixed pseudotyping, while a compatible combination is required for phenotypically mixed pseudotyping [218,223]. Moreover, we illustrate currently applied pseudotyping apporaches for HIV-1-derived lentiviral vectors in Figure 7B.

Regarding envelope proteins from *Retroviridae* family viruses, HIV-1 envelope protein gp120-gp41 binds the CD4 receptor and co-receptors (CCR5 and CXCR4), infecting CD4+ cells, allowing for HIV-1 investigation or gene delivery to target cells [211,225]. Murine leukemia virus amphotropic (MLV-A) envelope proteins (4070A, which recognizes Pit2 and 10A1 binds both Pit1 and Pit2) and murine leukemia virus ecotropic (MLV-E) envelope protein bind CAT1, thus expanding tropism in murine and human cells [226,227,228,229]. Gibbon Ape Leukemia Virus envelope protein (GALV) targets GLVR-1, and its pseudotyping efficiently transduces human T and B cells [230]. RD114 is a feline endogenous retrovirus-derived envelope protein that targets the ASCT2 receptor and confers high infectivity in CD34+ cells [231]. Avian sarcoma leukosis virus (ASLV) envelope proteins allow for the effective transduction of lentiviral vectors to neurons via recognizing TVA and TVB receptors [232]. Jaagsiekte sheep retrovirus (JSRV) envelope protein can bind glycosylphosphatidylinositol (GPI)-anchored Hyaluronidase 2 (Hyal2), and its pseudotyping is suitable for lung epithelial cell transduction [233]. Modified foamy viral envelope protein has shown benefits for gene transfer efficiency in CD34+ cells [234]. While Baboon endogenous virus (BaEV)-envelope protein pseudotyped lentiviral vector was shown to be effective in cytokine-stimulated NK cells [235], Koala retrovirus (KoRV) envelope protein pseudotyping was predominant in freshly isolated immune cells [236]. In addition, the R peptide-truncated BaEV (BaEVRless) envelope protein pseudotyped lentiviral vector retained infectivity in human serum, and had a superior ability to transduce cytokine-unstimulated CD34+ HSCs cells by targeting both ASCT1 and ASCT2 receptors in the presence of RetroNectin (a transduction enhancer), offering potential for ex vivo gene delivery targeting CD34+ HSCs [237,238,239].

Regarding envelope proteins from *Rhabdoviridae* family viruses, the VSV-G envelope glycoprotein targets low-density lipoprotein receptor (LDL-R) and is the most popular pseudotyping protein, having been used in both research and clinical applications due to its broad tropism, efficient packaging, stability, and robustness [11,219,224]; furthermore, it is inactivated by human serum [240]. Moreover, the envelope glycoprotein G of viruses in this family—such as Rabies virus (RABV), Chandipura vesiculo virus (CNV), Piry vesiculo virus (PRV), Maraba virus (MARV), Cocal virus (COCV), and Mokola virus (MOKV)—can be used in lentiviral pseudotyping, providing neuronal target delivery, reduced immunogenicity, and increased specificity [229,241,242,243,244].

Regarding the envelope proteins from *Baculoviridae* family viruses, the Baculovirus GP64 envelope glycoprotein pseudotyping for lentiviral vectors is an alternative option to replace standard VSV-G pseudotyping in general usage, due to its reduced immunity, lower toxicity, high stability, and broad tropism [245,246,247]. Heparan sulfate proteoglycan receptors and lipid rafts are recognized potential targets of the GP64 envelope protein [248].

Regarding envelope proteins from *Paramyxovirinae* subfamily viruses (*Paramyxoviridae* family), the modified hemagglutinin (H) and fusion (F) glycoprotein phenotypically unmixed dual-pseudotyped lentiviral vectors (H/F-LV) of the measles virus are considered a promising tool to introduce transgenes into immune cells and unstimulated CD34+ HSCs [249,250,251,252]. The phenotypically unmixed dual-pseudotyped lentiviral vectors with Sendai virus-modified HN and truncated F glycoproteins are considered ideal tools for delivering transgenes into airway cells, which are currently being employed in clinical trials [147,148,149]. Moreover, lentiviral vectors can be pseudotyped with HN and F of the human parainfluenza virus [253,254]. Furthermore, while some highly pathogenic Nipah and Hendra viruses are restricted to biosafety level-4 (BSL-4), the henipavirus F and G envelope glycoprotein pseudotyped lentiviral vector system provides a practical and low-risk technique for virology and biomedical research [255]. The modified F and wild-type G envelope proteins of the Nipah virus incorporate efficiently into the lentiviral vector and target ephrinB2+ cells, serving as a promising in vivo or in vitro delivery platform [256].

Regarding envelope proteins from *Pneumovirinae* subfamily viruses (*Paramyxoviridae* family), although human respiratory syncytial virus (RSV) is contagious, causing infection in the lungs and respiratory tract [257], a phenotypically unmixed heterologous triple-pseudotyped lentiviral vector (SH/G/F-LV) using the RSV envelope proteins SH, G, and F is considered useful in RSV virology research [220].

Regarding envelope proteins from *Hepadnaviridae* family viruses, a phenotypically unmixed heterologous triple-pseudotyped lentiviral vector (L/M/S-LV) using the L, M, and S envelope proteins of hepatitis B virus (HBV) has shown potential for liver-specific gene therapy and helped to elucidate HBV’s attachment and entry mechanisms [222].

Regarding envelope proteins from *Orthomyxoviridae* family viruses, a lentiviral vector pseudotyped with Hemagglutinin (HA) and neuraminidase (NA) envelope proteins from influenza viruses is considered as an alternative and attractive source for the study of influenza virology, in order to evaluate neutralizing antibodies and viral entry [258,259].

Regarding envelope proteins from *Filoviridae* family viruses, the severe hemorrhagic fever outbreak caused by Ebola and Marburg viruses—both BSL-4 pathogens—can lead to organ failure and death [260,261,262]. Therefore, the utilization of lentiviral vectors pseudotyped with the GP1 and GP2 envelope proteins from these threatening pathogens provides safer tools, allowing for better understanding of the underlying viral pathogenesis and efficient gene delivery [229,263,264,265].

Regarding envelope proteins from *Coronaviridae* family viruses, spike (S) and E envelope proteins from the SARS-CoV-1, MERS-CoV, or SARS-CoV-2 viruses pseudotyped lentiviral vectors have greatly contributed to the investigation of their viral entrance mechanisms, the evaluation of neutralizing antibodies, and the development of therapeutic approaches or vaccines [266,267].

Regarding envelope proteins from *Togaviridae* family viruses, this family comprises a range of zoonotic viruses—including chikungunya virus (CHIKV), sindbis virus (SINV), ross river virus (RRV), and semliki forest virus (SFV)—which cause a variety of symptoms, ranging from fever and headache to rash [268,269,270,271,272]. The generation and usage of the lentiviral vectors pseudotyped with E1 and E2 envelope proteins from these viruses has led to a better understanding of their basic virology and the immune responses associated with infection [273,274,275,276,277].

Regarding envelope proteins from *Flaviviridae* family viruses, the viruses in this family consist of important human pathogens, including zika virus (ZIKV), dengue virus (DENV), yellow fever virus (RFV), hepatitis C (HCV), and Japanese encephalitis virus (JEV) [268,278,279,280]. Pseudotyped lentiviral vectors using the envelope glycoproteins E1 and E2 (e.g., ZIKV, HCV, JEV) have shown benefits in investigating the attachment and entrance of the pathogens, the development of vaccines, and alteration of tropism [281,282,283].

Regarding viruses belonging to the *Bunyavirales* order, some cause zoonotic diseases; such as Rift Valley Fever Virus (RVFV) from the *Phenuiviridae* family, Crimean-Congo Hemorrhagic Fever Virus (CCHFV) from the *Nairoviridae* family, and Andes virus (ANDV) from the *Hantaviridae* family [284,285,286]. Lentiviral vectors pseudotyped with the Gn and Gc glycoproteins from these viruses have been utilized to study them and develop associated vaccines and treatments [287,288,289]. Moreover, several viruses from *Arenaviridae* family (*Bunyavirales* order)—including Lassa virus (LASV), Lujo virus (LUJV), Junin virus (JUNV), Machupo virus (MACV), Guanarito virus (GTOV), Tacaribe virus (TCRV), Chapare virus (CHAPV), and Sabiá virus—are responsible for causing zoonotic diseases and are classified as BSL-3 and BSL-4 pathogens [262,290,291,292]. Hence, lentiviral vectors pseudotyped with GP1 and GP2 envelope proteins from these zoonotic viruses are considered precious tools in the biomedical area [291,293]. In addition, the envelope proteins of non-pathogenic *Arenaviridae* family viruses, including Lymphocytic choriomeningitis virus (LCMV) and Pichinde virus (PICV), have also been used to pseudotype lentiviral vectors for virology research and tropism-altering purposes [229,294,295,296].

Regarding envelope proteins fused with scFV or nanobodies, the use of engineered envelope proteins for the lentiviral vectors (e.g., VSV-G glycoprotein fused with scFV or nanobodies, and Sindbis E1 and E2 glycoprotein fused with scFV or nanobodies) has demonstrated highly specific target delivery both in vivo and in vitro [297,298,299,300]. The VSV-G envelope protein fused with anti-CD30 scFV pseudotyping targets CD30+ lymphocytes [301], while the VSV-G fused with anti-EGFR scFV pseudotyping targets EGFR+ tumor cells [302]; furthermore, with poloxamer-based adjuvants, higher infectivity to the target cells than typical VSV-G pseudotyped LV was observed, offering potential in terms of cancer and immunotherapy. Moreover, binding-defective and fusion-competent VSV-G fused with antigen-presenting cell (APC)-specific nanobody pseudotyped LV was shown to selectively infect dendritic cells and macrophages both in vitro and in situ, demonstrating higher target delivery when compared with the VSV-G pseudotyped LV [298]. The CD90-targeting lentiviral vector, pseudotyped with either binding-defective and fusion-competent VSV-G with anti-CD90 scFV or binding-defective and fusion-competent measles H/F with anti-CD90 scFV, achieved success with lower off-target effects for ex vivo delivery in true HSCs, laying the foundation for in vivo gene therapy [303]. Thus, engineered envelope proteins—obtained via scFV and Nanobody display technology—provide the primary advantage of precise targeting both in vivo and ex vivo, which offers benefits including diminished off-target effects when compared to most envelope proteins with broad natural tropism, thus advancing the potential of lentiviral vectors.

In summary, pseudotyped lentiviral vectors with phenotypically unmixed or mixed heterologous envelope glycoproteins and engineered envelope proteins provide several advantages, such as expanding or altering viral tropism, enhancing stability, improving transduction efficiency, specific target delivery, lower risk, and reduced immune responses, providing particualr benefits in the context of gene therapy, vaccine development, basic science, and virology research.

## 6. Conclusions

Viruses have an innate ability to replicate by transferring genetic information and proteins into cells. Among viral vector platforms with delivery ability, lentiviral vectors have demonstrated promising capabilities in clinical applications due to their capacity to integrate genetic information into host cells’ DNA with minimal side effects, generally outweighing their disadvantages. Ultimately, the development of next-generation vectors focused on the viral genome and pseudotyping with heterologous or engineered envelope glycoproteins have made such systems versatile and beneficial tools in various applications, emphasizing their significant potential.

## Figures and Tables

**Table 1:** Examples of phenotypically unmixed and mixed pseudotyping of lentiviral vectors.

Phenotype	Glycoproteins	Origin	Pseudotyping and Lentiviral Vectors		Refs.
Unmixed	VSV-G (V)	Vesicular Stomatitis Virus	Single	V-LV	[224]
	Fusion (F) Hemagglutinin-neuraminidase (HN)	Sendai virus	Dual	F/HN-SIV, F/HN-LV	[148,149]
	Hemagglutinin (H) Fusion (F)	Measles virus	Dual	H/F-LV	[221]
	Small hydrophobic protein (SH) Glycoprotein G (G) Fusion (F)	Respiratory syncytial virus	Triple	SH/G/F-LV	[220]
	Large surface protein (L) Medium surface protein (M) Small surface protein (S)	Hepatitis B virus	Triple	L/M/S-LV	[222]
Mixed	VSV-G (V) Fusion (F)	Vesicular Stomatitis Virus Sendai virus	Dual	V/F-LV	[217,218]
	VSV-G (V) Hemagglutinin-neuraminidase (HN)	Vesicular Stomatitis Virus Sendai virus	Dual	V/HN-LV	[217,218]
	VSV-G (V) Fusion (F) Hemagglutinin-neuraminidase (HN)	Vesicular Stomatitis Virus Sendai virus	Triple	V/F/HN-LV	[217,218]

## Data Availability

All data supporting this review article are available in the Supplementary Files linked above.

## Supplementary Materials

The following supporting information can be downloaded at: https://www.mdpi.com/article/10.3390/v17081036/s1, Table S1: Current gene therapy clinical trials employing lentiviral vectors for human diseases; Table S2: Regulatory approved gene therapy products at the end of 2024.

## Abbreviations

AAV	Adeno-associated virus
Ad	Adenovirus
ADA	Adenosine deaminase
ADA-SCID	Severe combined immunodeficiency due to the adenosine deaminase deficiency
AFP	Alpha-fetoprotein
AIDS	Acquired immunodeficiency syndrome
AMD	Age-related macular degeneration
ASO	Antisense oligonucleotides
BCMA	B-cell maturation antigen
CA	Capsid
CALD	Cerebral adrenoleukodystrophy
CAR	Chimeric antigen receptor
Cas	CRISPR-associated protein
CFTR	Cystic fibrosis membrane conductance regulator
CMV	Cytomegalovirus
cPPT/CTS	Central polypurine tract/central termination sequence
CRISPR	Clustered regularly interspaced short palindromic repeats
DNA	Deoxyribonucleic acid
EMA	European Medicines Agency
F	Fusion
FDA	Food and Drug Administration
HBB	Hemoglobin subunit beta
HCC	Hepatocellular carcinoma
HIV	Human immunodeficiency virus
HN	Hemagglutinin-neuraminidase
HSCs	Hematopoietic stem cells
HSV	Herpes Simplex Virus
IL2RG	Interleukin 2 receptor subunit gamma
IN	Integrase
LTR	Long terminal repeat
MA	Matrix
MAGE-A4	Melanoma antigen gene A4
MERS-CoV	Middle east respiratory syndrome coronavirus
MESO	Mesothelin
MHC	Major Histocompatibility Complex
MLD	Metachromatic leukodystrophy
MLV	Murine leukemia virus
mRNA	Messenger RNA
NC	Nucleocapsid
NY-ESO-1	New York esophageal squamous cell carcinoma
OTC	Ornithine transcarbamylase
PR	Protease
RNA	Ribonucleic acid
RRE	Rev response element
RT	Reverse transcriptase
SARS-CoV	Severe acute respiratory syndrome coronavirus
scFV	Single-chain variable fragment
SeV	Sendai virus
sgRNA	Single guide RNA
shRNA	Short hairpin RNA
siRNA	Small interfering RNA
SIN-LTR	Self-inactivating LTR
SIV	Simian immunodeficiency virus
ssRNA	Single-stranded RNA
TALEN	Transcription activator-like effector nuclease
TCR	T-cell receptor
TRE	Tetracycline response element
vgRNA	Viral genomic RNA
vRNA	Viral RNA
VSV-G	Vesicular stomatitis virus glycoprotein G
WAS	Wiskott–Aldrich Syndrome
WPRE	Woodchuck Hepatitis Virus Post-Transcriptional Regulatory Element
X-SCID	X-linked severe combined immunodeficiency
ZNF	Zinc-finger nuclease
