# Correction: Jargalsaikhan et al. The Era of Gene Therapy: The Advancement of Lentiviral Vectors and Their Pseudotyping. *Viruses* 2025, *17*, 1036

**DOI:** 10.3390/v17111511

**Published:** 2025-11-18

**Authors:** Bat-Erdene Jargalsaikhan, Masanaga Muto, Masatsugu Ema

**Affiliations:** 1Department of Stem Cells and Human Disease Models, Research Center for Animal Life Science, Shiga University of Medical Science, Seta, Tsukinowa-cho, Otsu 520-2192, Japan; mmuto@belle.shiga-med.ac.jp (M.M.); mema@belle.shiga-med.ac.jp (M.E.); 2Medical Genome Center, National Cerebral and Cardiovascular Center, 6-1 Kishibe-Shinmachi, Suita 564-8565, Japan; 3Institute for the Advanced Study of Human Biology (ASHBi), Kyoto University, Yoshida-Konoe-cho, Sakyo-ku, Kyoto 606-8501, Japan

In the original publication [1], Figure 5D, the Vector-Intron (VI) feature after 2KO was missing, and an unnecessary polyA (pA) signal was included in the transfer vector. Figure 5D has been modified to include the VI and the extra pA after the SIN-LTR has been removed. The correct Figure 5 appears below.

## Text Correction

Regarding the inclusion of the VI feature in the figure of TetraVecta fourth-generation lentiviral vector system, the authors have revised paragraph 6 in Section 4, “Current Issues and Development of Next-Generation Lentiviral Vectors,” as follows:

“Another novel platform, the TetraVecta System™—fourth-generation lentiviral vector system by Oxford Biomedica (UK) in 2023—is characterized by enhanced safety, quality, and large payload capacity, achieved by introducing up to four systematic modifications on the transfer plasmid [191–194]. Firstly, the 2KO modification at the major splicing donor (MSD) site, optimized MSD-inactivating sequences, along with a new class of vRNA enhancers based on modified U1 snRNA [192], provides unspliced viral genomic RNA (vgRNA) during production, as HIV-1 splicing at the SD site is dependent on U1 snRNA [195]. The RRE/rev-independent version of the TetraVecta fourth-generation system (illustrated in Figure 5D) that contains the 2KO and synthetic Vector-Intron (VI) features does not rely on the modified U1 snRNA. Instead, the vgRNA is produced by splicing out of the VI, which occurs nearly 100% of the time [194]. Secondly, viral sequences with the RRE element are removed and replaced with the VI, allowing approximately 1 kb of additional space for the payload. The system is Rev-independent and does not need a regulatory plasmid vector [194]. Optionally, a bacterial tryptophan RNA-binding attenuation protein (TRAP) binding sequence overlaps the transcriptional start site of the transgene, and co-expression of TRAP inhibits transgene translation during viral vector production, which is Transgene Repression in Vector production (TRiP) system [196,197]. This is beneficial for the production of lentiviral vectors in CAR-T cells or cytotoxic payloads. The last refinement is bidirectional poly A sequences (supA) at the SIN-LTR, which are 50-fold stronger than the previously used sequences, thus improving transcriptional insulation [191,193].”

## References

The above correction requests amending references 193–195. The correct references are listed below:

193.Farley, D.C.; Wright, J. Improved SIN-LTRs for Lentiviral Vectors. Patent WO2023062365A2, 12 October 2022.194.Farley, D.C.; Wright, J. Rev/RRE-independent Lentiviral Vectors. Patent WO2023062363A1, 12 October 2022.195.Singh, R.N.; Singh, N.N. A Novel Role of U1 snRNP: Splice Site Selection from a Distance. *Biochim. Biophys. Acta. Gene Regul. Mech*. **2019**, *1862*, 634–642. https://doi.org/10.1016/j.bbagrm.2019.04.004.

The authors state that the scientific conclusions are unaffected. This correction was approved by the Academic Editor. The original publication has also been updated.

## Figures and Tables

**Figure 5 viruses-17-01511-f005:**
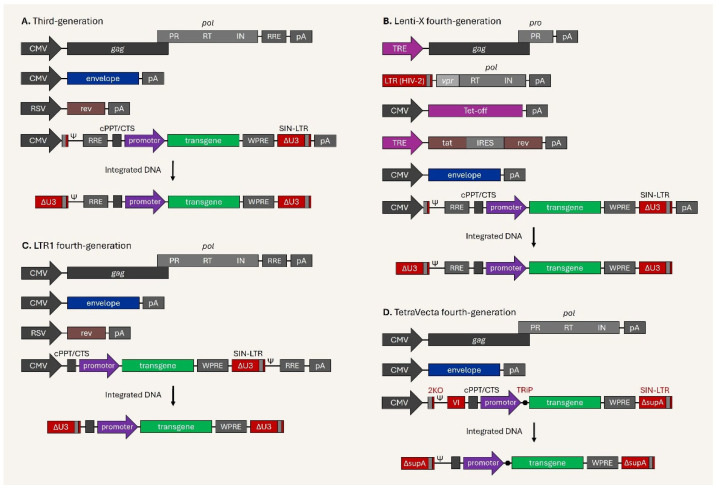
Schematic illustration of third-generation and next-generation lentiviral vectors. (**A**) Third-generation vector system with 4 plasmid vectors and integrated provirus. (**B**) Lenti-X fourth-generation vector system with 6 plasmid vectors and integrated provirus. (**C**) LTR1 fourth-generation vector system with 4 plasmid vectors and integrated provirus. (**D**) TetraVecta fourth-generation vector system with 3 plasmid vectors and integrated provirus.

## References

[1] Jargalsaikhan B.-E., Muto M., Ema M. (2025). The Era of Gene Therapy: The Advancement of Lentiviral Vectors and Their Pseudotyping. Viruses.

