# Supplementary material for: The Era of Gene Therapy: The Advancement of Lentiviral Vectors and Their Pseudotyping
Source: Viruses. 2025 Jul 24;17(8):1036. doi: 10.3390/v17081036 (PMC12390563; doi:10.3390/v17081036)
Supplement: Supplementary file 1 [file viruses-17-01036-s001.zip › Supplementary Table S2.pdf]

Supplementary Table S2. Regulatory approved gene therapy products by the end of 2024.

Brand and generic names	Developer or manufacturer	Approval	Type	Modification, packaged gene, or target	Mechanism of action	Indication	Ref
Vitravene (fomivirsen)	Ionis Pharmaceuticals	US FDA (1998)<br>EMA (1999)	ASO	A gapmer ASO with phosphorothioate linkage<br>- Targets UL123 mRNA	The ASO binds to the UL123 mRNA of cytomegalovirus (CMV) and inhibits translation.	Immunocompromised patients with CMV retinitis	[1,2]
Gendicine (rAd-p53)	Shenzhen SiBiono GeneTech	China FDA (2003)	Human adenovirus serotype 5 (Ad5)	- The Ad5 viral vector with the deletion of E1 and E3 region<br>- Packaged with human TP53 gene	Transferring the wild-type human TP53 gene into cancer cells inhibits the cells growth and induces apoptosis.	Head and neck squamous cell carcinoma	[3]
Macugen (pegaptanib)	EyeTech Pharmaceuticals	US FDA (2004)<br>EMA (2005)	ssRNA	Aptamer ssRNA with PEG moiety and with 2'-O-Me and 2'-F modifications<br>- Targets human VEGF protein	It binds to the isoform 165 of VEGF protein and inhibits its activity.	Neovascular age-related macular degeneration (AMD)	[4]
Oncorine (H101)	Shanghai Sunway Biotech	China FDA (2005)	Human adenovirus serotype 5 (Ad5)	The Ad5 viral vector with deletion of E1B-55kDa region and with partially deleted E3 region.	It selectively replicates and lyses cancer cells with dysfunctional p53 pathway.	- Head and neck nasopharyngeal carcinoma<br>- Esophageal squamous cell carcinoma	[5]
Rexin-G (mx-dnG1)	Epeius Biotechnologies	Philippine Bureau of Food and Drugs (2007)	Murine leukemia virus (MLV)	- Retrovirus pseudotyped with amphotropic envelope protein 4070A with collagen binding motif from vWF<br>- Packaged with dominant negative mutant cyclin G1 gene	Targeting abnormal signature proteins in tumors, and delivering the mutant cyclin G1 gene, causes cell cycle arrest at the G1 phase and induces apoptosis in the cancer cells.	Metastatic solid tumors<br>- Pancreatic cancer<br>- Breast cancer<br>- Osteosarcoma	[6]
Neovasculgen (pl-VEGF165)	Human Stem Cells Institute	Russian Ministry of Health (2011)	Plasmid DNA	Supercoiled plasmid DNA encoding VEGF165 protein	Delivering the plasmids into the target cells allows the translation of the VEGF165 protein, and stimulates angiogenesis in the surrounding area.	- Peripheral Arterial Disease (PAD)<br>- Critical Limb Ischemia (CLI)	[7]
Glybera (alipogene tiparvovec)	Amsterdam Molecular Therapeutics	European Commission (2012)	Adeno associated virus serotype 1 (AAV1)	Packaged with human lipoprotein lipase (LPL) gene variant LPL^{S447X}	Delivery and expression of a functional copy of the LPL gene to target cells, particularly muscle cells.	Familial lipoprotein lipase deficiency (LPLD)	[8]
Kynamro (mipomersen)	Ionis Pharmaceuticals	US FDA (2013)	ASO	A gapmer ASO with phosphorothioate linkage and 5mC modifications<br>- targets ApoB-100 mRNA	It binds to ApoB-100 mRNA and induces its degradation via RNase H, thereby reducing LDL cholesterol levels in the blood.	Homozygous familial hypercholesterolemia (HoFH)	[9]
Imlygic (talimogene laherparepvec)	Amgen, Inc.	EMA (2015)<br>US FDA (2017)	Human Herpes simplex virus 1 (HSV-1)	Oncolytic virus, deletions in the γ34.5 and α47 regions<br>- Packaged with human GM-CSF gene	It selectively replicates and lyses cancer cells and enhances the immune response against the tumor by translating GM-GSF.	Multiple solid tumors<br>- Melanoma<br>- Pancreatic Cancer	[10]
Exondys 51 (eteplirsen)	Sarepta Therapeutics	US FDA (2016)	ASO	Phosphorodiamidate morpholino oligomer (PMO)<br>- Targets dystrophin pre-mRNA	It binds exon 51 of dystrophin pre-mRNA, induces skipping the exon	Duchenne muscular dystrophy (DMD)	[2,11]

					during mRNA maturation, and allows translation of the functional protein.		
Spinraza (nusinersen)	Ionis Pharmaceuticals and Biogen	US FDA (2016) EMA (2017)	ASO	Phosphorothioate linkage and 5mC modification<br>- Targets SMN2 pre-mRNA	Binds to the intron 7 of the SMN2 pre-mRNA, allow proper splicing, which enhances the translation of functional SMN2 protein.	Spinal muscular atrophy (SMA) in children and adults	[12]
Strimvelis (autologous CD34+ enriched cell fraction)	Fondazione Telethon ETS	EMA (2016)	Autologous HSCs	Ex vivo gene genetically modified autologous HSCs transduced with MLV-based retroviral vector carrying the ADA gene	Intravenous infusion of the genetically modified HSCs and their engraftment in the body improves immune cell function by maintaining the production of the ADA protein.	Severe combined immunodeficiency due to adenosine deaminase deficiency (ADA-SCID)	[13]
Zalmoxis (allogeneic T cells genetically modified with a retroviral vector)	MolMed SPA	EMA (2016)	Allogeneic T cells	Ex vivo genetically modified allogenic T cells transduced with MLV-based retroviral vector carrying HSV-TK suicide and human ΔLNGFR marker gene	The expression of the HSV-TK suicide gene allows selective destruction of the infused allogeneic T cells by the administration of ganciclovir or valganciclovir.	Hematopoietic Stem Cell Transplantation Graft vs Host Disease	[14]
Ampligen (rintatolimod)	AIM Immunotech	Argentinean ANMAT (2016)	dsRNA	Mismatched dsRNA with phosphodiester linkage,<br>Poly I: Poly (C12U)<br>- Targets TLR3 protein	The dsRNA binds to the TLR3 receptor, triggering TLR3 activation, boosting the antiviral immune response.	Chronic fatigue syndrome/myalgic encephalomyelitis (CFS/ME)	[15]
Luxturna (voretigene neparvovec)	Spark Therapeutics, Inc.	US FDA (2017) EMA (2018)	Adeno associated virus serotype 2 (AAV2)	Packaged with normal copy of the human RPE65 gene	Delivery of the normal copy of the RPE65 gene restores vision by enabling the function of light-sensitive cells in the retina.	Inherited Retinal Dystrophies (IRD) associated with RPE65 gene mutations	[16]
Kymriah (tisagenlecleucel)	Novartis Pharmaceuticals Corporation	US FDA (2017) EMA (2018)	Autologous CAR-T cells	Ex vivo genetically modified autologous T cells transduced with a HIV-1-based lentiviral vector carrying anti-CD19 CAR	The T cells with an anti-CD19-scFv linked to signaling domains identify and eliminate cancerous B cells.	- Acute lymphoblastic leukemia (ALL)<br>- Diffuse large B-cell lymphoma (DLBCL)	[17]
Yescarta (axicabtagene ciloleucel)	Kite Pharma Inc.	US FDA (2017) EMA (2018) China NMPA (2021)	Autologous CAR-T cells	Ex vivo genetically modified autologous T cells transduced with a MSCV-based retroviral vector carrying anti-CD19 CAR	The T cells with an anti-CD19-scFv linked to signaling domains identify and eliminate cancerous B cells.	Relapsed or refractory large B-cell lymphoma (r/r LBCL)	[18]
Invossa (TissueGene-C)	Kolon Life Science and TissueGene, Inc.	South Korean MFDS (2017)	Allogeneic human chondrocytes	Ex vivo genetically modified allogeneic human chondrocytes transduced with MLV-based retroviral vector carrying the human TGF-β1 gene.	The TGF-β1 gene is expressed by the implanted chondrocytes into the affected joint represses inflammation and stimulates cartilage regeneration.	Knee Osteoarthritis	[19]
Onpattro (patisiran)	Alnylam Pharmaceuticals , Inc.	US FDA (2018) EMA (2018)	siRNA	Phosphodiester linkage with 2’-O-Me, 2’-dThd modifications, which is encapsulated in a lipid nanoparticle<br>- Targets TTR mRNA	It binds to TTR mRNA, triggering its degradation by the RISC complex. This results in decreased production, circulation, and deposition of TTR protein in tissues.	Hereditary transthyretin-mediated amyloidosis (hATTR amyloidosis)	[20]

Tegsedi (inotersen)	Akcea Therapeutics and Ionis Pharmaceuticals	US FDA (2018) EMA (2018)	ASO	A gapmer ASO with phosphorothioate linkage and 2'-O-MOE modifications - Targets TTR mRNA	It binds to TTR mRNA induces its degradation via RNase H, and reduces TTR protein translation.	Hereditary transthyretin-mediated amyloidosis (hATTR amyloidosis)	[2,21]
Zolgensma (onasemnogene abeparvovec-xioi)	Novartis Pharmaceuticals Corporation	US FDA (2019) EMA (2020)	Adeno associated virus serotype 9 (AAV9)	Packaged with normal copy of human the SMN1 gene	Delivery of the normal copy of the SMN1 gene to the motor neurons enables translation of the functional protein, helps to preserve neuron function, and prevents muscle degeneration.	Spinal muscular atrophy (SMA)	[22]
Collategene (beperminogene perplasmid)	AnGes	Japanese MHLW (2019)	Plasmid DNA	Plasmid DNA carrying the gene for human HGF (Hepatocyte Growth Factor)	Delivering the plasmids into the target cells allows the translation of the HGF protein, stimulates the growth and development of new blood vessels, thereby restoring blood circulation.	Critical Limb Ischemia	[23]
Gilvaari (Givosiran)	Alnylam Pharmaceuticals , Inc.	US FDA (2019)	siRNA	GalNAc conjugated siRNA with phosphodiester and phosphorothioate linkage, and with 2'-O-Me, and 2'-F modifications - Targets ALAS1 mRNA	It triggers degradation of the target mRNA by RISC complex, mainly in hepatocytes, resulting in reduced production of neurotoxic porphyrin precursors such as aminolaevulinic acid (ALA) and porphobilinogen (PBG), which prevents acute hepatic porphyria (AHP) attacks.	Adults with acute hepatic porphyria (AHP)	[24]
Vyondys 53 (golodirsen)	Sarepta Therapeutics	US FDA (2019)	ASO	Phosphorodiamidate morpholino oligomer (PMO) - Targets dystrophin pre-mRNA	It binds exon 53 of dystrophin pre-mRNA, induces skipping the exon during mRNA maturation, and allows translation of the functional protein.	Duchenne muscular dystrophy (DMD)	[25]
Waylivra (volanesorsen)	Ionis Pharmaceuticals	EMA (2019)	ASO	A gapmer ASO with phosphorothioate linkage and 2'-O-MOE modifications - Targets ApoC-III mRNA	It binds to ApoC-III mRNA, induce its degradation via RNase H, restraining the translation of the ApoC-III protein.	Familial chylomicronemia syndrome (FCS)	[26]
Zynteglo (betibeglogene autotemcel)	Bluebird bio-Inc.	EMA (2019) FDA (2022)	Autologous HSCs	Ex vivo gene genetically modified autologous HSCs transduced with HIV-1-based lentiviral vector carrying human β-globin gene ($\beta^{A\text{-}T87Q}$)	Intravenous infusion of the genetically modified HSCs and their engraftment in the body maintain the production of the β-globin (βA-T87Q) in erythroid cells.	Transfusion-dependent β thalassemia (TDT)	[27]
Viltepso (viltolarsen)	NS Pharma, Inc.	Japanese MHLW (2020) FDA (2020)	ASO	Phosphorodiamidate morpholino oligomer (PMO) - Targets dystrophin pre-mRNA	It binds exon 53 of dystrophin pre-mRNA, induces skipping the exon during mRNA maturation, and allows translation of the functional protein.	Duchenne muscular dystrophy (DMD)	[28]
Tecartus (brexucabtagene autoleucel)	Kite Pharmaceuticals , Inc.	US FDA (2020) EMA (2020)	CAR-T	Ex vivo genetically modified autologous T cells transduced with a	The T cells with an anti-CD19-scFv linked to signaling domains identify and eliminate cancerous B cells.	Relapsed or refractory mantle cell lymphoma (MCL)	[29]

				MSCV-based retroviral vector carrying anti-CD19 CAR			
Libmeldy/Lenmeldy (atidarsagene autotemcel)	Orchard Therapeutics	EMA (2020)<br>US FDA (2024)	Autologous HSCs	Ex vivo genetically modified autologous HSCs transduced with a HIV-1-based lentiviral vector carrying human ARSA gene.	Intravenous infusion of the genetically modified HSCs and their engraftment in the body maintain production of the functional ARSA protein, which reduces the accumulation of toxic sulfatide.	Metachromatic leukodystrophy (MLD)	[30]
Oxlumo (lumasiran)	Alnylam Pharmaceuticals, Inc.	US FDA (2020)	siRNA	GalNAc conjugated siRNA with phosphodiester and phosphorothioate linkage, and with 2'-O-Me, and 2'-F modifications<br>- Targets HAO1 mRNA	It triggers degradation of the target mRNA by RISC complex, resulting in reduced hepatic oxalate production by depleting glycolate oxidase	Primary hyperoxaluria type 1 (PH1)	[31]
Amondys 45 (casimersen)	Sarepta Therapeutics	US FDA (2021)	ASO	Phosphorodiamidate morpholino oligomer (PMO)<br>- Targets dystrophin pre-mRNA	It binds exon 45 of dystrophin pre-mRNA, induces skipping the exon during mRNA maturation, and allows translation of the functional protein.	Duchenne muscular dystrophy (DMD)	[32]
Breyanzi (lisocabtagene maraleucel)	Bristol-Myers Squibb	US FDA (2021)<br>EMA (2022)	Autologous CAR-T cells	Ex vivo genetically modified autologous T cells transduced with HIV-1-based lentiviral vector carrying anti-CD19 CAR	The T cells with an anti-CD19-scFv linked to signaling domains identify and eliminate cancerous B cells.	- Relapsed or refractory large B-cell lymphoma (r/r LBCL)<br>- Follicular lymphoma	[33]
Abecma (idecabtagene vicleucel)	Celgene Corporation, a Bristol-Myers Squibb Company	US FDA (2021)<br>EMA (2021)	Autologous CAR-T cells	Ex vivo genetically modified autologous T cells transduced with HIV-1-based lentiviral vector carrying anti-BCMA CAR	The T cells with an anti-BCMA-scFv linked to signaling domains identify and eliminate cancerous B cells.	Relapsed or refractory multiple myeloma (r/r MM)	[34]
Leqvio (inclisiran)	Alnylam Pharmaceuticals, Inc.	US FDA (2021)	siRNA	GalNAc conjugated siRNA with phosphodiester and phosphorothioate linkage, and with 2'-O-Me, and 2'-F modifications<br>- Targets PCSK9 mRNA	It triggers degradation of the target mRNA by RISC complex, resulting in reduced LDL cholesterol levels in the blood by inhibiting hepatic PCSK9 production	Primary hypercholesterolemia	[2,35]
Delytact (teserpaturev/G47Δ)	Daiichi Sankyo company	Japanese MHLW (2021)	Human herpes simplex virus 1 (HSV-1)	HSV-1 oncolytic virus with deletions in the γ34.5 and α47 regions, and insertion of LacZ gene in the ICP66 locus	It selectively infects and replicates within cancer cells, leading to their destruction by direct oncolysis	Malignant glioma	[36]
Relma-cel (relmacabtagene autoleucel)	JW Therapeutics	China National Medical Products Administration (2021)	Autologous CAR-T cells	Ex vivo genetically modified autologous T cells transduced with HIV-1-based lentiviral vector carrying anti-CD19 CAR	The T cells with an anti-CD19-scFv linked to signaling domains identify and eliminate cancerous B cells.	Relapsed or refractory large B-cell lymphoma (r/r LBCL)	[37]
Amvuttra (vutrisiran)	Alnylam Pharmaceuticals, Inc.	US FDA (2022)<br>EMA (2022)	siRNA	GalNAc conjugated siRNA with phosphodiester and phosphorothioate linkage, and with 2'-O-Me, and 2'-F modifications	It binds to TTR mRNA, triggering its degradation by the RISC complex. This results in decreased TTR production, mainly in liver.	Hereditary transthyretin-mediated amyloidosis (hATTR amyloidosis)	[38]

				- Targets TTR mRNA			
Skysona (elivaldogene autotemcel)	Bluebird bio-Inc.	US FDA (2022)	Autologous HSCs	Ex vivo genetically modified autologous HSCs transduced with a HIV-1-based lentiviral vector carrying human ABCD1 gene.	Intravenous infusion of genetically modified HSCs and their engraftment in the body leads to improve production of ALDP protein in the brain.	Cerebral Adreno Leukodystrophy (CALD)	[39]
Upstaza/Kebilidi (eladocagene exuparvovec-tneq)	PTC Therapeutics	EMA (2022)<br>US FDA (2024)	Adeno associated virus serotype 2 (AAV2)	Packaged with DDC gene under CMV promoter.	Delivery of the viral vector to brain (putamen region) restores functional AADC expression and leads to the development of motor function.	AADC deficiency	[40]
Adstiladrin (nadofaragene firadenovec-vncg)	Ferring Pharmaceuticals A/S	US FDA (2022)	Human adenovirus serotype 5 (Ad5)	The viral vector with the deletion of E1 and E3 region<br>- Packaged with human IFNα-2b gene	Upon local administration, the viral vector infects the urothelium with the assistance of Syn-3, encodes IFNα-2b, and shows immunostimulatory, antiangiogenic, and apoptotic effects against cancer.	Non-muscle invasive bladder cancer (NMIBC)	[41]
Carvykti (ciltacabtagene autoleucel)	Janssen Biotech, Inc.	US FDA (2022)<br>EMA (2022)	Autologous CAR-T cells	Ex vivo genetically modified autologous T cells transduced with HIV-1-based lentiviral vector carrying anti-BCMA CAR	The T cells with an anti-BCMA-scFv linked to signaling domains identify and eliminate cancerous B cells.	Relapsed or refractory multiple myeloma (r/r MM)	[42]
Hemgenix (etranacogene dezaparvovec-drlb)	CSL Behring LLC	US FDA (2022)<br>EMA (2023)	Adeno associated virus serotype 5 (AAV5)	Packaged with a high-activity variant of human coagulation factor IX gene (FIX-R338L) under liver specific promoter.	Upon intravenous administration, the viral vector targets liver cells, encoding FIX-R338L, reestablishing procoagulant activity and improves hemostasis.	Hemophilia B	[43]
Roctavian (valoctocogene roxaparvovec-rvox)	BioMarin Pharmaceutical Inc.	EMA (2022)<br>US FDA (2023)	Adeno associated virus serotype 5 (AAV5)	Packaged with a human coagulation factor VIII (FVIII) gene under liver specific promoter.	Upon intravenous administration, the viral vector targets liver cells, encoding FVIII, reestablishing procoagulant activity and improves hemostasis.	Hemophilia A	[44]
Lyfgenia (lovotibeglogene autotemcel)	Bluebird bio-Inc.	US FDA (2023)	Autologous HSCs	Ex vivo gene genetically modified autologous HSCs transduced with HIV-1-based lentiviral vector carrying human β-globin gene (βA-T87Q)	Intravenous infusion of the genetically modified HSCs and their engraftment in the body maintain the production of the β-globin (βA-T87Q) in erythroid cells.	Sickle cell disease (SCD)	[45]
Casgevy (exagamglogene autotemcel)	Vertex Pharmaceuticals Incorporated	US FDA (2023)<br>EMA (2024)	CRISPR modified autologous HSCs therapy	Ex vivo gene genetically modified autologous HSCs with CRISPR/Cas9 technology at the enhancer region of BCL11A gene, which disrupted GATA1 binding site.	Intravenous infusion of the modified HSCs and their engraftment in the body reduce BCL11A expression in erythroid cells, leading to increased HbF protein production.	- Sickle cell disease (SCD)<br>- Transfusion-dependent β-thalassemia (TDT)	[46]
Vyjuvek (beremagene geperpavec)	Krystal Biotech, Inc.	US FDA (2023)	Human herpes simplex virus 1 (HSV-1)	Replication-defective virus packaged with human functional COL7A1 genes	Upon topical administration to the wounds, the viral vectors enter skin cells, encoding COL7A1, producing COL7, which forms anchoring fibrils for wound healing.	Dystrophic epidermolysis bullosa (DEB)	[47]

Rivfloza (nedosiran)	Novo Nordisk, Inc.	US FDA (2023)	siRNA	GalNAc conjugated siRNA with phosphodiester and phosphorothioate linkage, and with 2'-O-Me, and 2'-F modifications<br>- Targets LDHA mRNA	It binds to LDHA mRNA, triggering its degradation by the RISC complex. This results in decreased hepatic LDH production.	Primary hyperoxaluria type 1 (PH1)	[48]
Qalsody (tofersen)	Ionis Pharmaceuticals and Biogen	US FDA (2023)<br>EMA (2024)	ASO	A gapmer ASO with phosphodiester and phosphorothioate linkage and with 2'-O-MOE modifications<br>- targets SOD1 mRNA	It binds to SOD1 mRNA and triggers its degradation via RNase H, reducing translation of the mutant SOD1 protein.	SOD1-amyotrophic lateral sclerosis (ALS)	[49]
Elevidys (delandistrogene moxeparvovec-rokl)	Sarepta Therapeutics, Inc.	US FDA (2023)	Adeno associated virus serotype rh74 (AAVrh74)	Packaged with shortened version of dystrophin protein gene	Upon intravenous administration, the viral vector targets muscle cells, encoding shortened functional dystrophin protein and helps mitigate muscle degeneration.	Duchenne muscular dystrophy (DMD)	[50]
Yuanruida (inaticabtagene autoleucel)	CASI Pharmaceuticals and Juventas Cell Therapy	Chinese NMPA (2023)	Autologous CAR-T cells	Ex vivo genetically modified autologous T cells transduced with HIV-1-based lentiviral vector carrying anti-CD19 CAR	The T cells with an anti-CD19-scFv linked to signaling domains identify and eliminate cancerous B cells.	Relapsed or refractory B-cell acute lymphoblastic leukemia (r/r B-ALL)	[51]
Fucaso (Equecabtagene autoleucel)	IASO Biotherapeutics and Innovent Biologics	Chinese NMPA (2023)	Autologous CAR-T cells	Ex vivo genetically modified autologous T cells transduced with HIV-1-based lentiviral vector carrying anti-BCMA CAR	The T cells with an anti-BCMA-scFv linked to signaling domains identify and eliminate cancerous B cells.	Relapsed or refractory multiple myeloma (r/r MM)	[52]
Saikaize (zevorcabtagene autoleucel)	Carsgen Therapeutics	Chinese NMPA (2024)	Autologous CAR-T cells	Ex vivo genetically modified autologous T cells transduced with HIV-1-based lentiviral vector carrying anti-BCMA CAR	The T cells with an anti-BCMA-scFv linked to signaling domains identify and eliminate cancerous B cells.	Relapsed or refractory multiple myeloma (r/r MM)	[53]
Tecelra (afamitresgene autoleucel)	Adaptimmune LLC	US FDA (2024)	Autologous TCR-T cells	Ex vivo genetically modified autologous T cells transduced with HIV-1-based lentiviral vector carrying TCR for human MAGE-A4.	The TCR-T cells identify cancer cells with MAGE-A4/HLA-A02 and destroy them by releasing cytokines.	Unresectable or metastatic synovial sarcoma	[54]
Beqvez (fidanacogene elaparvovec-dzkt)	Pfizer, Inc.	US FDA (2024)	Adeno associated virus serotype rh74 (AAVrh74)	Packaged with a high-activity variant of human coagulation factor IX gene (FIX-R338L) under liver specific promoter.	Upon intravenous administration, the viral vector targets liver cells, encoding FIX-R338L, reestablishing procoagulant activity and improves hemostasis.	Hemophilia B	[55]
Aucatzyl (obecabtagene autoleucel)	Autolus Limited	US FDA (2024)	Autologous CAR-T cells	Ex vivo genetically modified autologous T cells transduced with HIV-1-based lentiviral vector carrying anti-CD19 CAR.	The T cells with an anti-CD19-scFv linked to signaling domains identify and eliminate cancerous B cells.	Relapsed or refractory B-cell precursor acute lymphoblastic leukemia (r/r B-ALL)	[56]

References:

1. Vitravene Study Group A Randomized Controlled Clinical Trial of Intravitreous Fomivirsen for Treatment of Newly Diagnosed Peripheral Cytomegalovirus Retinitis in Patients with AIDS. *Am. J. Ophthalmol.* **2002**, *133*, 467–474, doi:10.1016/s0002-9394(02)01327-2.
2. Barresi, V.; Musmeci, C.; Rinaldi, A.; Condorelli, D.F. Transcript-Targeted Therapy Based on RNA Interference and Antisense Oligonucleotides: Current Applications and Novel Molecular Targets. *Int. J. Mol. Sci.* **2022**, *23*, doi:10.3390/ijms23168875.
3. Zhang, W.-W.; Li, L.; Li, D.; Liu, J.; Li, X.; Li, W.; Xu, X.; Zhang, M.J.; Chandler, L.A.; Lin, H.; et al. The First Approved Gene Therapy Product for Cancer Ad-P53 (Gendicine): 12 Years in the Clinic. *Hum. Gene Ther.* **2018**, *29*, 160–179, doi:10.1089/hum.2017.218.
4. Ng, E.W.M.; Shima, D.T.; Calias, P.; Cunningham, E.T.; Guyer, D.R.; Adamis, A.P. Pegaptanib, a Targeted Anti-VEGF Aptamer for Ocular Vascular Disease. *Nat. Rev. Drug Discov.* **2006**, *5*, 123–132, doi:10.1038/nrd1955.
5. Mondal, M.; Guo, J.; He, P.; Zhou, D. Recent Advances of Oncolytic Virus in Cancer Therapy. *Hum. Vaccin. Immunother.* **2020**, *16*, 2389–2402, doi:10.1080/21645515.2020.1723363.
6. Chawla, S.P.; Bruckner, H.; Morse, M.A.; Assudani, N.; Hall, F.L.; Gordon, E.M. A Phase I-II Study Using Rexin-G Tumor-Targeted Retrovector Encoding a Dominant-Negative Cyclin G1 Inhibitor for Advanced Pancreatic Cancer. *Mol. Ther. - Oncolytics* **2019**, *12*, 56–67, doi:10.1016/j.omto.2018.12.005.
7. Deev, R.; Plaksa, I.; Bozo, I.; Mzhavanadze, N.; Suchkov, I.; Chervyakov, Y.; Staroverov, I.; Kalinin, R.; Isaev, A. Results of 5-Year Follow-up Study in Patients with Peripheral Artery Disease Treated with PL-VEGF165 for Intermittent Claudication. *Ther. Adv. Cardiovasc. Dis.* **2018**, *12*, 237–246, doi:10.1177/1753944718786926.
8. Bryant, L.M.; Christopher, D.M.; Giles, A.R.; Hinderer, C.; Rodriguez, J.L.; Smith, J.B.; Traxler, E.A.; Tycko, J.; Wojno, A.P.; Wilson, J.M. Lessons Learned from the Clinical Development and Market Authorization of Glybera. *Hum. Gene Ther. Clin. Dev.* **2013**, *24*, 55–64, doi:10.1089/humc.2013.087.
9. Geary, R.S.; Baker, B.F.; Crooke, S.T. Clinical and Preclinical Pharmacokinetics and Pharmacodynamics of Mipomersen (Kynamro(®)): A Second-Generation Antisense Oligonucleotide Inhibitor of Apolipoprotein B. *Clin. Pharmacokinet.* **2015**, *54*, 133–146, doi:10.1007/s40262-014-0224-4.
10. Ferrucci, P.F.; Pala, L.; Conforti, F.; Cocorocchio, E. Talimogene Laherparepvec (T-VEC): An Intralesional Cancer Immunotherapy for Advanced Melanoma. *Cancers (Basel).* **2021**, *13*, 1383, doi:10.3390/cancers13061383.
11. Lim, K.R.Q.; Maruyama, R.; Yokota, T. Eteplirsen in the Treatment of Duchenne Muscular Dystrophy. *Drug Des. Devel. Ther.* **2017**, *11*, 533–545, doi:10.2147/DDDT.S97635.
12. Sumner, C.J.; Crawford, T.O. Two Breakthrough Gene-Targeted Treatments for Spinal Muscular Atrophy: Challenges Remain. *J. Clin. Invest.* **2018**, *128*, 3219–3227, doi:10.1172/JCI121658.
13. Migliavacca, M.; Barzaghi, F.; Fossati, C.; Rancoita, P.M. V.; Gabaldo, M.; Dionisio, F.; Giannelli, S.; Salerio, F.A.; Ferrua, F.; Tucci, F.; et al. Long-Term and Real-World Safety and Efficacy of Retroviral Gene Therapy for Adenosine Deaminase Deficiency. *Nat. Med.* **2024**, *30*, 488–497, doi:10.1038/s41591-023-02789-4.
14. Mohty, M.; Labopin, M.; Velardi, A.; van Lint, M.T.; Bunjes, D.; Bruno, B.; Santarone, S.; Tischer, J.; Koc, Y.; Wu, D.; et al. Allogeneic Genetically Modified T Cells (HSV-TK) As Adjunctive Treatment in Haploidentical Hematopoietic Stem-Cell Transplantation (Haplo-HSCT) of Adult Patients with High-Risk Hematological Malignancies: A Pair-Matched Analysis from the Acute Leukemia Wo. *Blood* **2016**, *128*, 672–672, doi:10.1182/blood.V128.22.672.672.
15. Mitchell, W.M. Efficacy of Rintatolimod in the Treatment of Chronic Fatigue Syndrome/Myalgic Encephalomyelitis (CFS/ME). *Expert Rev. Clin.*

Pharmacol. **2016**, *9*, 755–770, doi:10.1586/17512433.2016.1172960.
16. Testa, F.; Bacci, G.; Falsini, B.; Iarossi, G.; Melillo, P.; Mucciolo, D.P.; Murro, V.; Salvetti, A.P.; Sodi, A.; Staurenghi, G.; et al. Voretigene Neparvovec for Inherited Retinal Dystrophy Due to RPE65 Mutations: A Scoping Review of Eligibility and Treatment Challenges from Clinical Trials to Real Practice. *Eye (Lond).* **2024**, *38*, 2504–2515, doi:10.1038/s41433-024-03065-6.
17. Schuster, S.J.; Bishop, M.R.; Tam, C.S.; Waller, E.K.; Borchmann, P.; McGuirk, J.P.; Jäger, U.; Jaglowski, S.; Andreadis, C.; Westin, J.R.; et al. Tisagenlecleucel in Adult Relapsed or Refractory Diffuse Large B-Cell Lymphoma. *N. Engl. J. Med.* **2019**, *380*, 45–56, doi:10.1056/NEJMoa1804980.
18. Locke, F.L.; Miklos, D.B.; Jacobson, C.A.; Perales, M.-A.; Kersten, M.-J.; Oluwole, O.O.; Ghobadi, A.; Rapoport, A.P.; McGuirk, J.; Pagel, J.M.; et al. Axicabtagene Ciloleucel as Second-Line Therapy for Large B-Cell Lymphoma. *N. Engl. J. Med.* **2022**, *386*, 640–654, doi:10.1056/NEJMoa2116133.
19. Kim, M.-K.; Ha, C.-W.; In, Y.; Cho, S.-D.; Choi, E.-S.; Ha, J.-K.; Lee, J.-H.; Yoo, J.-D.; Bin, S.-I.; Choi, C.-H.; et al. A Multicenter, Double-Blind, Phase III Clinical Trial to Evaluate the Efficacy and Safety of a Cell and Gene Therapy in Knee Osteoarthritis Patients. *Hum. Gene Ther. Clin. Dev.* **2018**, *29*, 48–59, doi:10.1089/humc.2017.249.
20. Maurer, M.S.; Kale, P.; Fontana, M.; Berk, J.L.; Grogan, M.; Gustafsson, F.; Hung, R.R.; Gottlieb, R.L.; Damy, T.; González-Duarte, A.; et al. Patisiran Treatment in Patients with Transthyretin Cardiac Amyloidosis. *N. Engl. J. Med.* **2023**, *389*, 1553–1565, doi:10.1056/NEJMoa2300757.
21. Benson, M.D.; Waddington-Cruz, M.; Berk, J.L.; Polydefkis, M.; Dyck, P.J.; Wang, A.K.; Planté-Bordeneuve, V.; Barroso, F.A.; Merlini, G.; Obici, L.; et al. Inotersen Treatment for Patients with Hereditary Transthyretin Amyloidosis. *N. Engl. J. Med.* **2018**, *379*, 22–31, doi:10.1056/NEJMoa1716793.
22. Strauss, K.A.; Farrar, M.A.; Muntoni, F.; Saito, K.; Mendell, J.R.; Servais, L.; McMillan, H.J.; Finkel, R.S.; Swoboda, K.J.; Kwon, J.M.; et al. Onasemnogene Abeparvovec for Presymptomatic Infants with Two Copies of SMN2 at Risk for Spinal Muscular Atrophy Type 1: The Phase III SPR1NT Trial. *Nat. Med.* **2022**, *28*, 1381–1389, doi:10.1038/s41591-022-01866-4.
23. Komatsuno, T. The First Gene Therapy Product in Japan Collategene® Intramuscular Injection. *Drug Deliv. Syst.* **2019**, *34*, 305–308, doi:10.2745/dds.34.305.
24. Dickey, A.K.; Leaf, R.K. Givosiran: A Targeted Treatment for Acute Intermittent Porphyria. *Hematol. Am. Soc. Hematol. Educ. Progr.* **2024**, *2024*, 426–433, doi:10.1182/hematology.2024000663.
25. Scaglioni, D.; Catapano, F.; Ellis, M.; Torelli, S.; Chambers, D.; Feng, L.; Beck, M.; Sewry, C.; Monforte, M.; Harriman, S.; et al. The Administration of Antisense Oligonucleotide Golodirsen Reduces Pathological Regeneration in Patients with Duchenne Muscular Dystrophy. *Acta Neuropathol. Commun.* **2021**, *9*, 7, doi:10.1186/s40478-020-01106-1.
26. Witztum, J.L.; Gaudet, D.; Freedman, S.D.; Alexander, V.J.; Digenio, A.; Williams, K.R.; Yang, Q.; Hughes, S.G.; Geary, R.S.; Arca, M.; et al. Volanesorsen and Triglyceride Levels in Familial Chylomicronemia Syndrome. *N. Engl. J. Med.* **2019**, *381*, 531–542, doi:10.1056/NEJMoa1715944.
27. Locatelli, F.; Thompson, A.A.; Kwiatkowski, J.L.; Porter, J.B.; Thrasher, A.J.; Hongeng, S.; Sauer, M.G.; Thuret, I.; Lal, A.; Algeri, M.; et al. Betibeglogene Autotemcel Gene Therapy for Non–β 0 /β 0 Genotype β-Thalassemia. *N. Engl. J. Med.* **2022**, *386*, 415–427, doi:10.1056/NEJMoa2113206.
28. Roshmi, R.R.; Yokota, T. Pharmacological Profile of Viltolarsen for the Treatment of Duchenne Muscular Dystrophy: A Japanese Experience. *Clin. Pharmacol.* **2021**, *13*, 235–242, doi:10.2147/CPAA.S288842.
29. Wang, Y.; Jain, P.; Locke, F.L.; Maurer, M.J.; Frank, M.J.; Munoz, J.L.; Dahiya, S.; Beitinjaneh, A.M.; Jacobs, M.T.; Mcguirk, J.P.; et al. Brexucabtagene Autoleucel for Relapsed or Refractory Mantle Cell Lymphoma in Standard-of-Care Practice: Results From the US Lymphoma CAR T Consortium. *J. Clin. Oncol.* **2023**, *41*, 2594–2606, doi:10.1200/JCO.22.01797.
30. Fumagalli, F.; Calbi, V.; Natali Sora, M.G.; Sessa, M.; Baldoli, C.; Rancoita, P.M. V; Ciotti, F.; Sarzana, M.; Fraschini, M.; Zambon, A.A.; et al. Lentiviral

Haematopoietic Stem-Cell Gene Therapy for Early-Onset Metachromatic Leukodystrophy: Long-Term Results from a Non-Randomised, Open-Label, Phase 1/2 Trial and Expanded Access. *Lancet (London, England)* **2022**, *399*, 372–383, doi:10.1016/S0140-6736(21)02017-1.
31. Garrelfs, S.F.; Frishberg, Y.; Hulton, S.A.; Koren, M.J.; O'Riordan, W.D.; Cochat, P.; Deschênes, G.; Shasha-Lavsky, H.; Saland, J.M.; Van't Hoff, W.G.; et al. Lumasiran, an RNAi Therapeutic for Primary Hyperoxaluria Type 1. *N. Engl. J. Med.* **2021**, *384*, 1216–1226, doi:10.1056/NEJMoa2021712.
32. Wagner, K.R.; Kuntz, N.L.; Koenig, E.; East, L.; Upadhyay, S.; Han, B.; Shieh, P.B. Safety, Tolerability, and Pharmacokinetics of Casimersen in Patients with Duchenne Muscular Dystrophy Amenable to Exon 45 Skipping: A Randomized, Double-Blind, Placebo-Controlled, Dose-Titration Trial. *Muscle Nerve* **2021**, *64*, 285–292, doi:10.1002/mus.27347.
33. Abramson, J.S.; Solomon, S.R.; Arnason, J.; Johnston, P.B.; Glass, B.; Bachanova, V.; Ibrahimi, S.; Mielke, S.; Mutsaers, P.; Hernandez-Ilizaliturri, F.; et al. Lisocabtagene Maraleucel as Second-Line Therapy for Large B-Cell Lymphoma: Primary Analysis of the Phase 3 TRANSFORM Study. *Blood* **2023**, *141*, 1675–1684, doi:10.1182/blood.2022018730.
34. Munshi, N.C.; Anderson, L.D.; Shah, N.; Madduri, D.; Berdeja, J.; Lonial, S.; Raje, N.; Lin, Y.; Siegel, D.; Oriol, A.; et al. Idecabtagene Vicleucel in Relapsed and Refractory Multiple Myeloma. *N. Engl. J. Med.* **2021**, *384*, 705–716, doi:10.1056/NEJMoa2024850.
35. Ray, K.K.; Troquay, R.P.T.; Visseren, F.L.J.; Leiter, L.A.; Scott Wright, R.; Vikarunnessa, S.; Talloczy, Z.; Zang, X.; Maheux, P.; Lesogor, A.; et al. Long-Term Efficacy and Safety of Inclisiran in Patients with High Cardiovascular Risk and Elevated LDL Cholesterol (ORION-3): Results from the 4-Year Open-Label Extension of the ORION-1 Trial. *lancet. Diabetes Endocrinol.* **2023**, *11*, 109–119, doi:10.1016/S2213-8587(22)00353-9.
36. Frampton, J.E. Teserpaturev/G47Δ: First Approval. *BioDrugs* **2022**, *36*, 667–672, doi:10.1007/s40259-022-00553-7.
37. Ying, Z.; Yang, H.; Guo, Y.; Li, W.; Zou, D.; Zhou, D.; Wang, Z.; Zhang, M.; Wu, J.; Liu, H.; et al. Relmacabtagene Autoleucel (Relma-Cel) CD19 CAR-T Therapy for Adults with Heavily Pretreated Relapsed/Refractory Large B-Cell Lymphoma in China. *Cancer Med.* **2021**, *10*, 999–1011, doi:10.1002/cam4.3686.
38. Fontana, M.; Berk, J.L.; Gillmore, J.D.; Witteles, R.M.; Grogan, M.; Drachman, B.; Damy, T.; Garcia-Pavia, P.; Taubel, J.; Solomon, S.D.; et al. Vutrisiran in Patients with Transthyretin Amyloidosis with Cardiomyopathy. *N. Engl. J. Med.* **2025**, *392*, 33–44, doi:10.1056/NEJMoa2409134.
39. Eichler, F.; Duncan, C.N.; Musolino, P.L.; Lund, T.C.; Gupta, A.O.; De Oliveira, S.; Thrasher, A.J.; Aubourg, P.; Kühl, J.-S.; Loes, D.J.; et al. Lentiviral Gene Therapy for Cerebral Adrenoleukodystrophy. *N. Engl. J. Med.* **2024**, *391*, 1302–1312, doi:10.1056/NEJMoa2400442.
40. Tai, C.-H.; Lee, N.-C.; Chien, Y.-H.; Byrne, B.J.; Muramatsu, S.-I.; Tseng, S.-H.; Hwu, W.-L. Long-Term Efficacy and Safety of Eladocagene Exuparvovec in Patients with AADC Deficiency. *Mol. Ther.* **2022**, *30*, 509–518, doi:10.1016/j.ymthe.2021.11.005.
41. Lee, A. Nadofaragene Firadenovec: First Approval. *Drugs* **2023**, *83*, 353–357, doi:10.1007/s40265-023-01846-z.
42. Martin, T.; Usmani, S.Z.; Berdeja, J.G.; Agha, M.; Cohen, A.D.; Hari, P.; Avigan, D.; Deol, A.; Htut, M.; Lesokhin, A.; et al. Ciltacabtagene Autoleucel, an Anti-B-Cell Maturation Antigen Chimeric Antigen Receptor T-Cell Therapy, for Relapsed/Refractory Multiple Myeloma: CARTITUDE-1 2-Year Follow-Up. *J. Clin. Oncol.* **2023**, *41*, 1265–1274, doi:10.1200/JCO.22.00842.
43. Pipe, S.W.; Leebeek, F.W.G.; Recht, M.; Key, N.S.; Castaman, G.; Miesbach, W.; Lattimore, S.; Peerlinck, K.; Van der Valk, P.; Coppens, M.; et al. Gene Therapy with Etranacogene Dezaparvovec for Hemophilia B. *N. Engl. J. Med.* **2023**, *388*, 706–718, doi:10.1056/NEJMoa2211644.
44. Ozelo, M.C.; Mahlangu, J.; Pasi, K.J.; Giermasz, A.; Leavitt, A.D.; Laffan, M.; Symington, E.; Quon, D. V.; Wang, J.-D.; Peerlinck, K.; et al. Valoctocogene Roxaparvovec Gene Therapy for Hemophilia A. *N. Engl. J. Med.* **2022**, *386*, 1013–1025, doi:10.1056/NEJMoa2113708.
45. Kanter, J.; Chawla, A.; Thompson, A.A.; Kwiatkowski, J.L.; Parikh, S.; Mapara, M.Y.; Rifkin-Zenenberg, S.; Aygun, B.; Kasow, K.A.; Gupta, A.O.; et al. Lovotibeglogene Autotemcel Gene Therapy for Sickle Cell Disease: 60 Months Follow-Up. *J. Sick. Cell Dis.* **2024**, *1*, doi:10.1093/jscdis/yoae002.002.

46. Frangoul, H.; Locatelli, F.; Sharma, A.; Bhatia, M.; Mapara, M.; Molinari, L.; Wall, D.; Liem, R.I.; Telfer, P.; Shah, A.J.; et al. Exagamglogene Autotemcel for Severe Sickle Cell Disease. *N. Engl. J. Med.* **2024**, *390*, 1649–1662, doi:10.1056/NEJMoa2309676.
47. Dhillon, S. Beremagene Geperpavec: First Approval. *Drugs* **2023**, *83*, 1131–1135, doi:10.1007/s40265-023-01921-5.
48. Syed, Y.Y. Nedosiran: First Approval. *Drugs* **2023**, *83*, 1729–1733, doi:10.1007/s40265-023-01976-4.
49. Wiesenfarth, M.; Dorst, J.; Brenner, D.; Elmas, Z.; Parlak, Ö.; Uzelac, Z.; Kandler, K.; Mayer, K.; Weiland, U.; Herrmann, C.; et al. Effects of Tofersen Treatment in Patients with SOD1-ALS in a "Real-World" Setting - a 12-Month Multicenter Cohort Study from the German Early Access Program. *EClinicalMedicine* **2024**, *69*, 102495, doi:10.1016/j.eclinm.2024.102495.
50. Hoy, S.M. Delandistrogene Moxeparvovec: First Approval. *Drugs* **2023**, *83*, 1323–1329, doi:10.1007/s40265-023-01929-x.
51. Wang, Y.; Lv, L.; Song, Y.; Wei, X.; Zhou, H.; Liu, Q.; Xu, K.; Yan, D.; Zhang, C.; Liu, S.; et al. Inaticabtagene Autoleucel in Adult Relapsed or Refractory B-Cell Acute Lymphoblastic Leukemia. *Blood Adv.* **2025**, *9*, 836–843, doi:10.1182/bloodadvances.2024014182.
52. Keam, S.J. Equecabtagene Autoleucel: First Approval. *Mol. Diagn. Ther.* **2023**, *27*, 781–787, doi:10.1007/s40291-023-00673-y.
53. Dhillon, S. Zevorcabtagene Autoleucel: First Approval. *Mol. Diagn. Ther.* **2024**, *28*, 501–506, doi:10.1007/s40291-024-00723-z.
54. D'Angelo, S.P.; Araujo, D.M.; Abdul Razak, A.R.; Agulnik, M.; Attia, S.; Blay, J.-Y.; Carrasco Garcia, I.; Charlson, J.A.; Choy, E.; Demetri, G.D.; et al. Afamitresgene Autoleucel for Advanced Synovial Sarcoma and Myxoid Round Cell Liposarcoma (SPEARHEAD-1): An International, Open-Label, Phase 2 Trial. *Lancet* **2024**, *403*, 1460–1471, doi:10.1016/S0140-6736(24)00319-2.
55. Cuker, A.; Kavakli, K.; Frenzel, L.; Wang, J.-D.; Astermark, J.; Cerqueira, M.H.; Iorio, A.; Katsarou-Fasouli, O.; Klamroth, R.; Shapiro, A.D.; et al. Gene Therapy with Fidanacogene Elaparvovec in Adults with Hemophilia B. *N. Engl. J. Med.* **2024**, *391*, 1108–1118, doi:10.1056/NEJMoa2302982.
56. Roddie, C.; Sandhu, K.S.; Tholouli, E.; Logan, A.C.; Shaughnessy, P.; Barba, P.; Ghobadi, A.; Guerreiro, M.; Yallop, D.; Abedi, M.; et al. Obecabtagene Autoleucel in Adults with B-Cell Acute Lymphoblastic Leukemia. *N. Engl. J. Med.* **2024**, *391*, 2219–2230, doi:10.1056/NEJMoa2406526.
